# Quantitatively Different, yet Qualitatively Alike: A Meta-Analysis of the Mouse Core Gut Microbiome with a View towards the Human Gut Microbiome

**DOI:** 10.1371/journal.pone.0062578

**Published:** 2013-05-01

**Authors:** Lukasz Krych, Camilla H. F. Hansen, Axel K. Hansen, Frans W. J. van den Berg, Dennis S. Nielsen

**Affiliations:** 1 Department of Food Science, Faculty of Science, University of Copenhagen, Copenhagen, Denmark; 2 Department of Veterinary Disease Biology, Faculty of Health and Medical Sciences, University of Copenhagen, Copenhagen, Denmark; Charité-University Medicine Berlin, Germany

## Abstract

**Background:**

A number of human diseases such as obesity and diabetes are associated with changes or imbalances in the gut microbiota (GM). Laboratory mice are commonly used as experimental models for such disorders. The introduction and dynamic development of next generation sequencing techniques have enabled detailed mapping of the GM of both humans and animal models. Nevertheless there is still a significant knowledge gap regarding the human and mouse common GM core and thus the applicability of the latter as an animal model. The aim of the present study was to identify inter- and intra-individual differences and similarities between the GM composition of particular mouse strains and humans.

**Methodology/Principal Findings:**

A total of 1509428 high quality tag-encoded partial 16S rRNA gene sequences determined using 454/FLX Titanium (Roche) pyro-sequencing reflecting the GM composition of 32 human samples from 16 individuals and 88 mouse samples from three laboratory mouse strains commonly used in diabetes research were analyzed using Principal Coordinate Analysis (PCoA), nonparametric multivariate analysis of similarity (ANOSIM) and alpha diversity measures. A reliable cutoff threshold for low abundant taxa estimated on the basis of the present study is recommended for similar trials.

**Conclusions/Significance:**

Distinctive quantitative differences in the relative abundance of most taxonomic groups between the examined categories were found. All investigated mouse strains clustered separately, but with a range of shared features when compared to the human GM. However, both mouse fecal, caecal and human fecal samples shared to a large extent not only representatives of the same phyla, but also a substantial fraction of common genera, where the number of shared genera increased with sequencing depth. In conclusion, the GM of mice and humans is quantitatively different (in terms of abundance of specific phyla and species) but share a large qualitatively similar core.

## Introduction

Shifts in the composition, known as dysbiosis, of the human GM have in several studies been associated with diseases such as allergies [1], asthma [2], inflammatory bowel disease [3], diabetes type 1 and 2 [4]–[6], and metabolic syndrome [7] - all indicating a causative role of the gut microbiota.

Rodents are the mammalian model most extensively used to investigate the relationship between GM and health and disease. The reason for the popularity of mouse models is their well explored genetic and relatively close physiological similarity with humans and the ability to control a wide range of environmental factors which reduces variation in the baseline gut microbiota between individual study objects [8]. However, despite the wide use of rodent models existing information about the human and laboratory mouse common GM core is still relatively scarce. One of the pioneering reports on this field, carried out before the age of next-generation sequencing, disclosed only 15% similarity between the human and mouse GM genera [9]. The majority of bacteria in the gut were shown to be members of the two phyla, Firmicutes and Bacteroidetes, and in both humans and mice, these two phyla together comprised more than 90% of the gut bacteria.

Many GM related disorders have been linked with bacterial dysbiosis on a higher taxonomic level proving the usefulness of sequencing the GM to e.g. phylum and family level. For example, Turnbaugh et al. showed that a switch from a low fat to a high fat, high sugar diet in mice, which was associated with obesity, lowered the Bacteroidetes/Firmicutes ratio in the gut within one day [10]. Also, alterations in the phylum Bacteroidetes and the *Lachnospiraceae* family have been suggested as possible biomarkers to help predict predispositions to inflammatory bowel disease [11]. In humans, patients suffering from T2D have been found to have significant reductions in the phylum Firmicutes and the class Clostridia compared to healthy controls in a 454 FLX based study [5]. In a later study comparing the gut metagenome of individuals suffering from T2D and healthy controls a group of butyrate producing bacteria and opportunistic pathogens that could serve as gut microbial markers for classifying type 2 diabetes were identified [12] underlining the possibility of identifying microbial markers at this taxonomic level associated with disease. Despite the advantages of deep metagenome sequencing it still remains a costly approach and a range of reports show that the relationship between many disorders and GM changes can be identified without the need for studying whole metagenomes [5], [6], [11], [13], [14].

However, the usefulness of mice models for such studies would to a large extent also depend on similarities in their GM profiles at genus or species level with humans. The aim of the present study was therefore to demonstrate inter- and intra-individual differences and similarities between the GM composition of three laboratory mouse strains commonly used in research in chronic inflammatory diseases with those of humans based on more than 1.5 million high quality sequences of partial 16S rRNA gene verified with tag-encoded 454/FLX Titanium (Roche) pyro-sequencing.

## Materials and Methods

### Dataset

A total of 88 mice and 128 human (16 individuals with each individual sampled twice within 6 weeks and each sample sequenced 4 times) GM profiles determined using tag-encoded 16S rRNA gene 454/FLX Titanium (Roche) pyro-sequencing were included in the study (Table 1). All samples enrolled in the present meta-analysis have been treated according to the same protocols concerning DNA extraction, library preparation and sequencing [6], [14]. Briefly, cellular DNA was extracted using a QIAamp DNA Stool Mini Kit (Qia- gen, Hilden, Germany) basically following the manufacturer’s instructions, but with the addition of an initial bead beating step (FastPrep) for increasing cell lysis. Extracted DNA was stored at −40°C until analysis. Amplicons (466 bp) including the V3 and V4 regions of the 16S rRNA gene were amplified using the primers detailed in the electronic supplementary material (ESM) Table 1 [15] followed by a second round of PCR where primers with adapters and tags were used [16]. PCR amplification of the 16S rRNA gene plus purification and pyrosequencing of amplified PCR products were carried out as previously described [5]. The amplified fragments with adapters and tags were quantified using a Qubit fluorometer (Invitrogen, Carlsbad, CA, USA) and mixed in approximately equal concentrations to ensure most possible even representation of reads per sample. Two- region 454 sequencing runs were performed on a GS FLX Titanium Pico TiterPlates (70×75) using a GS FLX Titanium Sequencing Kit XLR70 according to the manufacturer’s instructions (Roche Diagnostics, Indianapolis, IN, USA).

**Table 1:** Data collection description.

NCBI accessionnumber	Host	Host’s age when sampled	Sample type	Number of samples	Total number of raw sequences	High quality reads					
						Total	Avg.	Max	Min	SD	Average sequence length (bp)
**SRA058021**	Human (control)	18–50 years	Feces	16×4	469971	392726	6136	10273	2582	2050	446
**SRA058021**	Human (placebo)	18–50 years	Feces	16×4	509407	402262	6385	11126	2296	2433	446
**SRA047328**	NOD	14–30 weeks	Feces	15	122164	118515	8465	15139	5668	3120	359
**SRA057283**	B6. V-*Lep ^ob^*/J	8 weeks	Feces	19	157688	150229	7907	10257	5641	1084	467
**SRA057283**	B6. V-*Lep ^ob^*/J	16 weeks	Feces	19	154484	142519	7501	9122	4169	1314	465
**SRA059019**	BALB/c	13 weeks	Feces	21	232612	203586	9695	13987	6030	2371	464
**SRA051317**	BALB/c	10 weeks	Caecumcontent	14	107532	99591	7114	8470	5667	888	450

Sequence collections representing five control groups from previously published studies used for this meta-analysis were stored in the Sequence Read Archive (SRA, http://www.ncbi.nlm.nih.gov/sra), National Center for Biotechnology Information (NCBI). The pool of human samples (SRA058021) composed of the control and the placebo group. Samples from these two categories were sequenced in four independent runs resulting in 16 human GM profiles sequenced in 4 replicates for each sample collected at time zero (the control group) and 4 replicates for samples collected after 6 weeks from the time zero (the placebo group). The B6. V-*Lep ^ob^*/J mice (SRA057283) composed of the two sampled at 8 and 16 weeks of age. The GM profile of BALB/c mice was verified for caecal and fecal samples (studies SRA051317 and SRA059019 respectively).

All animal experiments were carried out in accordance with the Council of Europe Convention European Treaty Series (ETS) 123 on the Protection of Vertebrate Animals used for Experimental and Other Scientific Purposes, and the Danish Animal Experimentation Act (LBK 1306 from 23/11/2007). The study was approved by the Animal Experiments Inspectorate, Ministry of Justice, Denmark.

Human specimens used in this meta-analysis come from the independent study that was approved by The Scientific Ethics Committee of Capital Region, Denmark (reference H-4-2010-137). Written informed consent was obtained from volunteers prior to recruitment.

### Data Treatment

The dataset was analyzed using the Quantitative Insight Into Microbial Ecology (QIIME) open source software package [17]. All steps such as quality control, de-noising, chimera filtering and OTU picking were conducted as previously described [14]. High quality sequences purged from chimeric reads were further clustered at 97% relatedness using UCLAST (http://www.drive5.com/usearch/). The representative sequences from each cluster were aligned with pyNAST (http://qiime.org/pynast/) and subjected to the Ribosomal Database Project (RDP)-based 16S rRNA gene annotation. For intra-individual assessment all mouse and 16 human samples from the control group were subsampled to an equal number of reads per individual (4500 reads per sample which constitutes to 85% of the second most indigent sample in the dataset). For inter-group comparison the reads were merged according to host (mouse strain/human) and subsampled to an equal number of sequences per category, respectively 80000 reads for mice strains comparisons and 600000 reads for similarity assessment between mice and humans. Both numbers constitute approximately 85% of the least numerous category. Alpha diversity measures such as rarefaction curves based on the estimated species number (97% sequence identity threshold), Chao1 and Shannon indexes were calculated for OTU tables that were unified to 4000 (first most indigent sample) sequences per sample.

In order to investigate the influence of the sequencing method into variance between categories, which is caused mostly by the low abundant taxa, a set of 16 samples each sequenced in 4 independent runs were compared within their quadruplicates. A set of 1000 subsampled OTU-tables was generated for each sample (3000 reads per sample). Low abundant taxa were removed until taxa similarity of all replicates within a given sample crossed 99%. An average, minimum cutoff value was therefore calculated based on 16000 subsampled OTU tables using an in-house Matlab (Mathworks) script.

### Statistics

Principal Coordinate Analysis (PCoA) plots were generated with the Jackknifed Beta Diversity workflow based on 10 distance metrics calculated using 10 subsampled OTU tables. The -e value (number of sequences taken for each jackknifed subset) was set to 85% of the sequence number within the most indigent sample. Analysis of similarities (ANOSIM) was used to evaluate group differences using weighted and unweighted uniFrac distance metrics that were generated based on rarefied (4500 reads per sample) OTU tables. The relative distribution of the GM genera registered in 88 mouse and 16 human samples was calculated for unified, summarized at the genus level OTU tables.

Differences in taxa abundances at phylum and genus level between categories were verified with Metastats (http://metastats.cbcb.umd.edu). From each group 14 samples (corresponding to the smallest category) were randomly chosen and combinations of group pairs were tested using 1000 permutations (p value threshold = 0.05; false discovery rate threshold = 0.5). The relationship between sequencing depth and shared GM, classified into phylum and genus level, between mice and humans was plotted based on multiple subsampled OTU tables composed of two categories collecting 794988 human and 714440 mouse GM 16S rRNA gene reads. Simulation of each sequencing depth was repeated 100 times and an average proportion of shared taxonomic groups between the two categories were calculated (abundance threshold for unshared taxa = 0.19%).

A network presenting shared genera between categories was prepared for normalized OTU tables (600000 reads per category) after filtering the low abundant OTUs (abundance threshold for unshared taxa = 0.19%) using the make_otu_network.py script (QIIME). The visualization of the OTU-networks was performed with an open source platform –Cytoscape (version 2.8.3, http://www.cytoscape.org/).

## Results

The number of sequences collected that fulfilled quality control requirements (minimum sequence length ≥250 bp, minimum average quality score ≥25) yielded 1753858. After removing chimeric sequences a total of 1509428 reads remained, meaning that the ChimeraSlayer algorithm [18] used for chimeras purging reduced the dataset with approximately 14%. Information about the total, average, maximum, minimum, and standard deviation of high quality, noise and chimera-free reads are collated in Table 1.

### Gut Microbiota Composition

**Figure 1:**
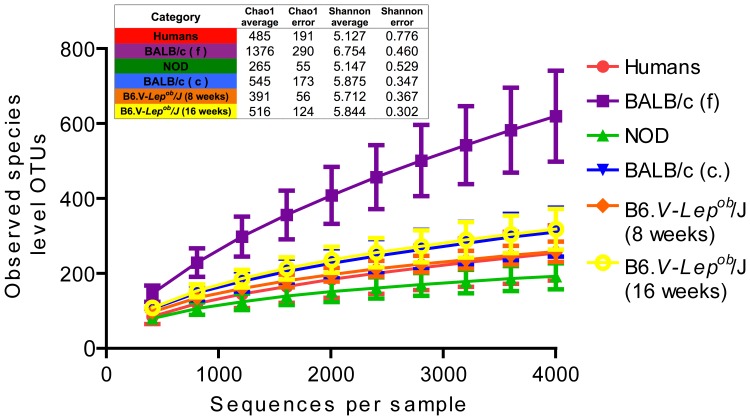
Rarefaction curves based on the estimated number of OTUs. (97% sequence identity threshold), average Cho1 and Shannon indexes were calculated for 6 categories using rarefied OTU table (4000 reads per sample). Labels “BALB/c (f)” and “BALB/c (c)” stand for the GM microbiota profile of BALB/c mice determined using fecal and caecal samples respectively.

Alpha diversity assessment using rarefaction curves revealed that the fecal GM of the BALB/c mice is the most rich in OTUs (species-level). The average Chao1 index calculated for 4000 reads per sample was 2.5–5 times higher in this group compared with other groups (Figure 1). Species-level OTUs entropy portrayed with the Shannon’s diversity index was correspondingly higher in the fecal samples of BALB/c mice (Figure 1).

**Figure 2:**
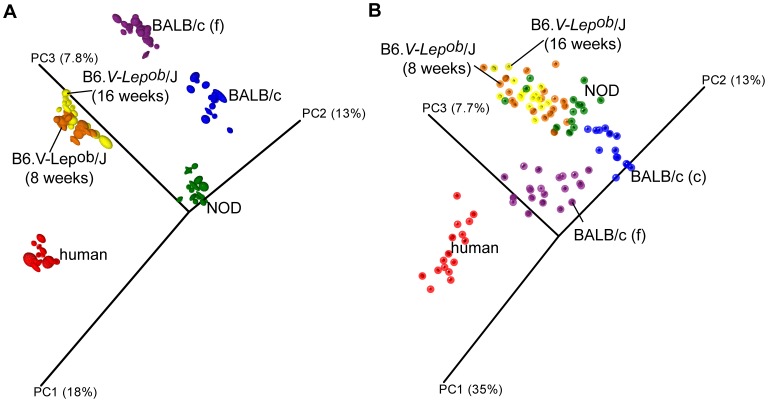
PCoA plot based on (A) unweighted and (B) weighted distance matrices, each calculated from 10 rarefied (4500 reads per sample) OTU tables. A) Qualitative information used to generate principal coordinates enables for clear clustering according to host that samples were collected from. B) Quantitative information used to generate principal coordinates enables for clear separation of human and both BALB/c samples and less distinct but significant (Table 2) separation of the NOD and two B6 mice GM profiles. Labels “BALB/c (f)” and “BALB/c (c)” stand for the gut GM of BALB/c mice determined using fecal and caecal samples respectively. The degree of variation between 10 jackknifed replicates of PCoA is displayed with confidence ellipsoids around each sample.

PCoA analysis based on unweighted UniFrac distance matrices showed a clear clustering of samples according to host (Figure 2A) with the human GM distinctly separated from the mice, as also shown by ANOSIM analysis (Table 2). The two B6. V-*Lep^ob^*/J groups where age was the only varying factor were only partially separated (Fig. 2A and Table 2). When widening the information with the bacterial relative abundance (weighted UniFrac distance matrix) the differences became less distinct, and the frontier between the GM of NOD and B6. V-*Lep^ob^*/J mice became less clear (Figure 2B) but ANOSIM analysis showed that the separation was still significant (Table 2, p<0.001***). As R values >0.75 generally are interpreted as clearly separated, R >0.5 as separated and R <0.25 as groups hardly separated [19] it was concluded that all categories with the exception of the two B6. V-*Lep^ob^*/J mouse groups could be classified as a clearly separated or separated.

**Table 2:** ANOSIM analysis between categories.

	B6 (8 weeks)		B6 (16 weeks)		BALB/C (feces)		BALB/C (caecum)		NOD		Human	
	R	p	R	p	R	p	R	p	R	p	R	p
**6. V-** ***Lep^ob^*** **/J** **(8 weeks)**	–	–	**0.644**	<0.001***	**1.000**	<0.001***	**0.999**	<0.001***	**1.000**	<0.001***	**1.000**	<0.001***
**B6. V-** ***Lep^ob^*** **/J (16 weeks)**	0.219	<0.001***	–	–	**1.000**	<0.001***	**0.998**	<0.001***	**0.998**	<0.001***	**1.000**	<0.001***
**BALB/c** **(feces)**	0.808	<0.001***	0.888	<0.001***	–	–	**0.883**	<0.001***	**0.999**	<0.001***	**1.000**	<0.001***
**BALB/c (caecum)**	0.663	<0.001***	0.876	<0.001***	0.850	<0.001***	–	–	**0.994**	<0.001***	**1.000**	<0.001***
**NOD**	0.531	<0.001***	0.564	<0.001***	0.901	<0.001***	0.475	<0.001***	–	–	**1.000**	<0.001***
**Human**	0.911	<0.001***	0.888	<0.001***	0.975	<0.001***	0.851	<0.001***	0.829	<0.001***	–	–

Analysis of similarity (testing whether two or more groups are significantly different) was calculated between all categories based on rarefied (4500 reads per sample) weighted (regular font) and unweighted (bold font) distance matrices. Each pairwise comparison of two groups was performed using 1000 permutations. R values >0.75 are generally interpreted as clearly separated, R >0.5 as separated and R <0.25 as groups hardly separated [17].

### Abundance Threshold

All samples in the human study SRA058021 have been sequenced four times in independent runs. However, when comparing the similarity within the same sample sequenced four times it was found that only an average of 77.1% of taxa was shared due to the nature of the sequencing method where low abundant taxa may or may not be captured by pure chance [20]. Starting with the assumption that all four replicates representing a given sample should depict roughly the same relative distribution of bacteria this dataset was then used to calculate the most commensurate cut off value for low abundant taxa, that were not a true picture of low abundant microbial groups but which presence or absence was rather a result of a sequencing method/depth. Consequently, low abundant reads were removed until all 4 replicates of a single sample shared at least 99% of taxa. Sixty-four (16×4) samples from the above-mentioned group were included and 100 subsampled OTU tables were generated for each replicate (3000 reads per sample). The threshold for the low abundant taxa that needed to be removed in order to make all 4 replicates uniform was evaluated based on 16000 rarefied OTU tables and scored: 0.19%, which corresponded to approximately 10 reads per taxon. When a certain taxon was registered in all replicates of a given sample and one or more were below the estimated threshold level, all values were kept.

### Phyla Distribution and Abundance

All reads used in this study were classified into 9 phyla after applying the abundance threshold (0.19%), with one phylum noted as unclassified (Table 3). Generally Firmicutes and Bacteroidetes were the dominating phyla accounting for 89–97% of all reads with a clear preponderance of Firmicutes in all categories except the BALB/c mice (Table 3). Verrucomicrobia was the third most abundant phyla in NOD mice and the fourth most abundant in humans and B6.V-*Lep^ob^*J (8 weeks of age). The three remaining mouse strains were either devoid of bacteria from this phylum, or the bacteria were below the detection limit.

**Table 3:** The relative distribution of phyla among categories.

	NOD				BALB/c (cecum)				B6 (16 weeks of age)				B6 (8 weeks of age)				BALB/c (feces)				Human			
	Avg.	Max	Min	SD	Avg.	Max	Min	SD	Avg.	Max	Min	SD	Avg.	Max	Min	SD	Avg.	Max	Min	SD	Avg.	Max	Min	SD
***Actinobacteria***	0.11	1.20	0.00	0.32	0.03	0.12	0.00	0.03	0.05	0.30	0.02	0.07	0.03	0.06	0.00	0.02	0.07	0.16	0.00	0.04	1.15	5.18	0.02	1.55
***Bacteroidetes***	22.47	45.40	7.36	12.09	22.07	34.40	11.40	7.55	32.85	54.50	20.72	9.14	35.54	58.54	19.24	12.14	51.37	71.18	20.54	11.89	16.73	26.08	2.94	7.16
***Deferribacteres***	0.48	2.84	0.06	0.69	1.06	2.28	0.06	0.66	0.19	0.54	0.00	0.14	0.14	0.82	0.00	0.22	0.12	0.52	0.00	0.17	0.00	0.00	0.00	0.00
***Firmicutes***	66.43	90.82	47.92	15.90	69.99	78.48	56.82	6.84	64.38	77.66	41.56	9.63	58.93	78.04	35.20	14.06	43.90	75.26	27.08	12.33	74.92	95.34	44.54	12.41
***Unclassified***	2.39	10.36	0.94	2.46	4.07	7.70	1.10	1.99	2.12	3.86	0.78	0.95	2.92	5.20	1.56	1.10	3.34	8.86	1.42	2.03	1.63	7.66	0.02	2.54
***Proteobacteria***	0.08	0.20	0.00	0.06	2.05	9.76	0.14	2.44	0.26	0.42	0.12	0.08	0.34	1.16	0.08	0.25	0.93	2.56	0.08	0.80	3.17	17.04	0.16	4.59
***TM7***	0.04	0.26	0.00	0.07	0.70	2.36	0.24	0.57	0.10	0.32	0.00	0.10	0.12	0.46	0.00	0.13	0.22	0.60	0.00	0.20	0.00	0.02	0.00	0.01
***Tenericutes***	0.29	1.16	0.00	0.38	0.02	0.10	0.00	0.03	0.02	0.10	0.00	0.03	0.09	0.58	0.00	0.15	0.01	0.04	0.00	0.01	0.87	13.32	0.00	3.32
***Verrucomicrobia***	7.69	31.90	0.04	11.23	0.00	0.00	0.00	0.00	0.00	0.00	0.00	0.00	1.85	30.24	0.00	6.90	0.00	0.00	0.00	0.00	1.51	13.76	0.00	3.76

The relative distribution of the gut microbial phyla among single human (16 individuals) and five mouse groups (15, 14, 19, 19, 21 mice respectively). The abundance threshold within at least one of the category was set to 0.19%.

### Genera Distribution and Abundance

**Figure 3:**
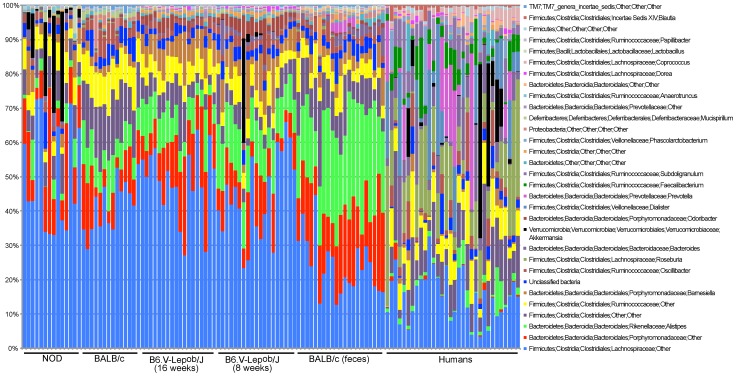
The relative distribution of the major genera (31) determined for rarefied (4500 reads per sample) 88 mouse and 32 human samples. Sequences annotation pursued using the Ribosomal Database Project (RDP, http://rdp.cme.msu.edu/) database.

The relative abundance of genera depicted for all samples individually shows explicit alterations between studies with the human specimens being the most conspicuous category (Figure 3). In total 239 genera were registered for both groups but only 89 exceeded the threshold value (Table S1). The unclassified genus from the Lachnospiraceae family was the most dominant bacterial group in both mouse and human samples comprising on average 41% and 15% of the reads, respectively. The two consecutive most abundant genera in mouse samples were an unclassified genus from the Porphyromonadaceae (13%) and *Alistipes* (12%), while in human specimens – *Roseburia* (12%) and unclassified genus representing the Clostridiales order (10%) were the dominating genera.

Metastats analysis revealed widespread differences in the bacterial relative abundance at both phylum (Table S2) and genus (Table S3) level between all categories.

### Gut Microbiota Qualitative Differences between Mice

**Figure 4:**
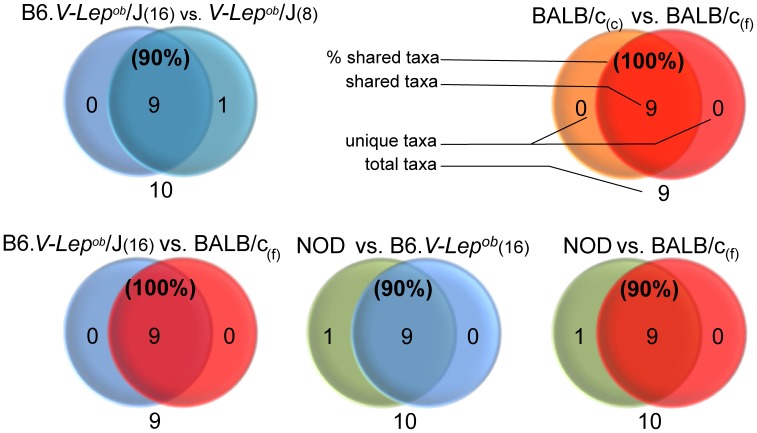
Number of GM phyla shared between given mouse strains after applying a cut off threshold (0.19%) for the low abundant taxa. Whenever a given genus was present in both categories but the value for one or both was bellow the threshold level the label was kept and classified as shared. 80000 high quality 16s rRNA reads used to represent each mouse strain were annotated to the Ribosomal Database Project (RDP, http://rdp.cme.msu.edu/) database. In three cases the single phylum Verrucomicrobia is reducing similarity to 90%. The only genus representing this subgroup is *Akkermansia*. Labels “BALB/c (f)”, “BALB/c (c)”, “B6.V-*Lep^ob^*/J (16)” and “B6.V-*Lep^ob^*/J (8)” stand for the gut GM of BALB/c mice determined using fecal and caecal samples and B6.V-*Lep^ob^*/J mice using fecal specimens sampled in 16 and 8 weeks of age respectively.

**Figure 5:**
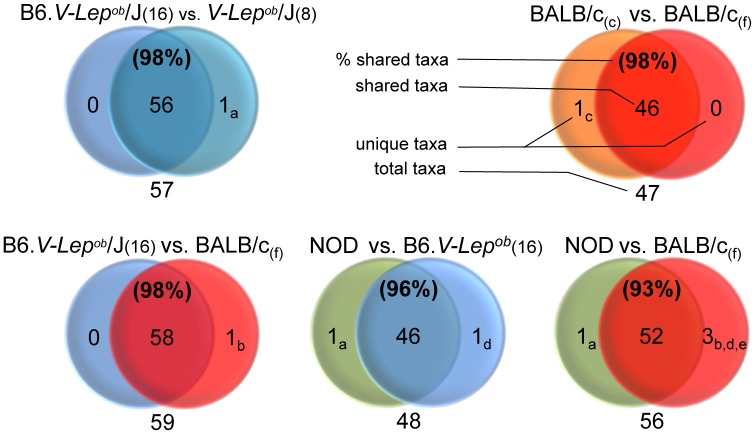
Number of GM genera shared between given mouse strains after applying a cut off threshold (0.19%) for the low abundant taxa. Whenever a given genus was present in both categories but the value for one or both was bellow the threshold level the label was kept and classified as shared. 80000 high quality 16s rRNA reads used to represent each mouse strain were annotated to the Ribosomal Database Project (RDP, http://rdp.cme.msu.edu/) database. Genera differing between categories: a -*Akkermansia*, b – *Prevotella*, c- unclassified genus from Desulfovibrionales order, d – *Odoribacter*, e – unclassified genus from Proteobacteria phylum (source: Table S1).

Despite ubiquitous quantitative differences in GM distribution between categories considerable qualitative similarities of the most abundant taxa were observed between all examined groups. To a large extent mouse fecal and caecal samples shared the same bacterial phyla (Figure 4) while on the genus level the five mouse groups shared between 93–98% of the generic labels (Figure 5). The Verrucomicrobia phylum present in the fecal microbiota of the B6. *V-Lep^ob^*/J pups (analyzed in the age of 8 weeks) and the NOD group was not detected in the adult B6. *V-Lep^ob^*/J counterparts (examined in the age of 16 weeks), nor in any of the BALB/c mice. The *Akkermansia* genus which is the only representative group of the Verrucomicrobia phylum, was therefore the reason of discrepancies on both the phylum (Figure 4) and the genus level (Figure 5) between categories. The unclassified genus from the Desulfovibrionales order present in the caecal content of the BALB/c mice was not found in the fecal specimens (Figure 5). However both categories shared 100% of the phyla, and likewise, the adult B6. *V-Lep^ob^*/J and the BALB/c mice were equal on the phylum level (Figure 4) albeit B6. *V-Lep^ob^*/J mice lacked members of the *Prevotella* genus when compared to the BALB/c mice. Furthermore, the NOD group was more indigent genus-vise lacking the *Odoribacter* genus compared to the B6. *V-Lep^ob^*/J and *Prevotella, Odoribacter* and unclassified genera from Proteobacteria phylum compared to the BALB/c mice (Figure 5).

### Gut Microbiota Qualitative Differences between Mice and Humans

**Figure 6:**
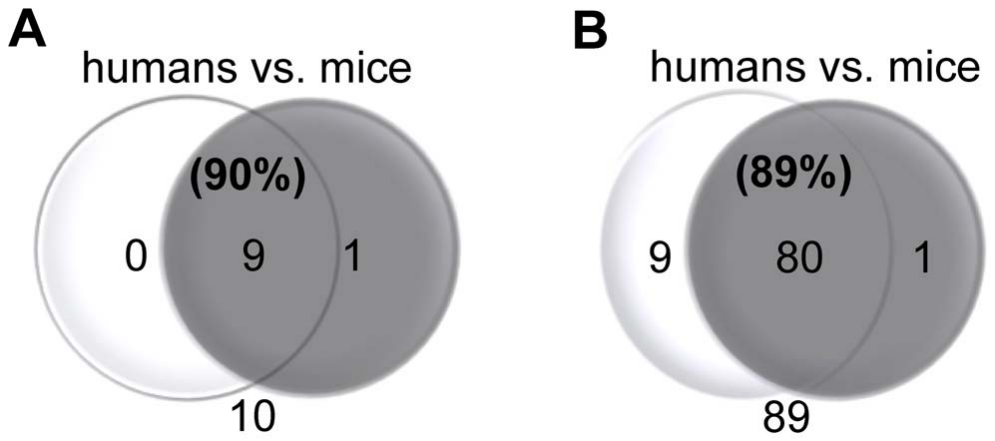
Number of GM taxa shared between collated categories of humans and mice on both, phylum (A) and genus (B) level. 600000 high quality 16s rRNA reads used to represent each category were annotated to the Ribosomal Database Project (RDP, http://rdp.cme.msu.edu/) database. A cut off threshold for the low abundant taxa = 0.19%. Whenever a given taxon was present in both categories but the value for one or both was bellow the threshold level the label was kept and classified as shared. **A)** Phylum Deferribacteres was unique for the mouse category what reduced similarity between categories to 90%. **B)** Genus *Mucispirillum* unique for mouse category was the only representative of phylum Deferribacteres. Human GM cluster contained 9 unique genera groups, namely *Faecalibacterium, Mitsuokellla, Megasphera, Dialister, Asteroleplasma, Succinivibio, Sutterella, Paraprevotella* and *Phascolarctobacterium* (source: Table S1).

Mice and humans shared 90% of bacterial phyla with Deferribacteres and its only genus deputy - *Mucispirillum* causing the difference between mouse and human categories at the qualitative level (Figure 6A). Analysis on a deeper classification level showed that above the threshold value mouse and human samples shared 89% of bacterial genera (Figure 6B). The human GM cluster contained 9 unique genera compared to the mice, namely *Faecalibacterium, Mitsuokellla, Megasphera, Dialister, Asteroleplasma, Succinivibio, Sutterella, Paraprevotella* and *Phascolarctobacterium*. The collection of mice GM profiles presented one unique genus, *Mucispirillum,* while the remaining 80 genera despite differences in the relative abundance were common for both mice and humans (Figure S1).

The average similarity of the GM phyla and genera between mice strains verified using the raw dataset, without the abundance threshold, was respectively 20% and 56% lower than when using a cutoff value (Figures S2A and S2B). The similarity of the collective mouse and human microbiomes without the abundance threshold was correspondingly 40% and 57% lower at the phylum and the genus level (Figure S2C) compared to the dataset where the threshold was employed.

**Figure 7:**
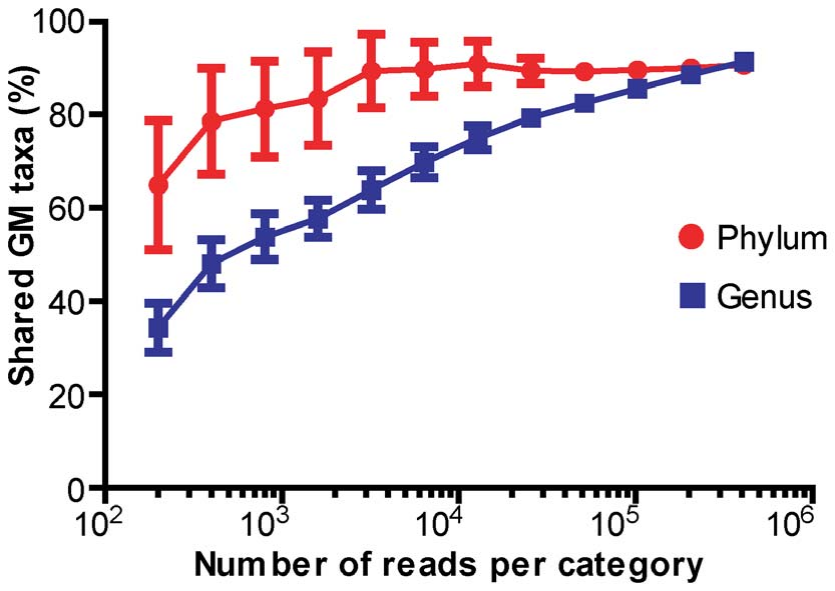
Function of sequencing depth and gut microbial taxa similarity between mice and humans. A set of 100 subsampled OTU-tables were generated for each simulated sequencing depth and further summarized into the phylum and the genus level. The plot presents how sequencing depth influences the qualitative similarity rate between mouse and human GM annotated into two distant phylogenetic levels. Although relatively shallow sequencing is enough to reach plateau at the phylum level, much deeper sequencing is required for inferring at the genus level taxa.

In Figure 7 the function between sequencing depth and GM similarity at the phylum and genus level is illustrated. It was found that both phyla and - even more pronounced - genera resemblance between categories differs dramatically depending on number of reads used for analysis as increasing sequencing depth uncovers more genera that the two groups have in common (Figure 7).

## Discussion

Laboratory mice are commonly used as experimental models for diseases such as diabetes [6], [21], inflammatory bowel disease [22], [23] and allergies [24], [25] where the GM composition and function has been found to be an important contributing factor [26]. However, at present a knowledge gap regarding the similarity of the human and mouse common GM core exists, especially on a deeper level of taxonomy, which might question the usefulness of these models. Therefore, in the present study, we analyzed inter- and intra-individual differences and similarities between the GM composition of three laboratory mouse strains commonly used in diabetes research with those of humans based on more than 1.5 million high quality sequences of partial 16S rRNA gene verified with tag-encoded 454/FLX Titanium (Roche) pyro-sequencing.

Qualitative and quantitative-based analysis of the three mouse strains fecal GM compared with the mice caecal and human fecal GM (PCoA and ANOSIM) disclosed significant separation of all microbial profiles. The composition of the GM has previously been shown to be influenced by multiple factors including environment and host genetics [8], [27]–[31]. The latter seems to be a major force driving the GM differences between the various mice and human clusters. It is therefore the genetic resemblance between different mouse strains that propagates similar bacterial groups to develop and at the same time keeps other ones at a low abundance. Consequently, despite ubiquitous species level differences, the bacterial relative distribution on higher taxonomic levels makes the GM of different mice strains more similar to each other compared to that of humans (Fig. 2 and 3).

Diet is an environmental factor known to strongly influence GM composition [32], [33]. However all mice used in this study were fed with a similar chow diet (Altromin 1324); therefore diet was the least differing factor of microbial community between mouse strains but definitely a strong one when comparing with the human GM. Diet is therefore another force in addition to genetics pushing human GM cluster away from the mice.

Early priming of bacterial colonization during early life may also influence GM and immunity later in life [34]–[38], which might also be a driving force in the present study. Especially the environment of humans is undoubtedly the most dissimilar from that of laboratory mice, what again favors receding human bacterial profile away from the mice. Consequently, these differences are important to consider when performing mice-human translational studies.

Lastly, for adult humans and mice the influence of age on the GM profiles seems to be minor as neither human samples collected from patients at different age (18–50 years) nor NOD mice analyzed at different ages (14–30 weeks) clustered according to age. This supports the high level of GM composition stability in adult individuals which was previously documented among humans and mice [27], [39]–[41].

Although innovations in high throughput sequencing techniques offer insight into microbial communities at an hitherto up-preceded level of details one need to be aware of constrains that comes with it, for example: sequencing depth, reads error (noise, chimeras) rate and length or analysis methods [42]. Furthermore, in the present study it was found that differences in sequencing depth between samples influences qualitative comparison between categories. The range of this effect could be estimated using the human samples included in the analysis that have been independently sequenced four times. When comparing independently sequenced data sets, representing the same fecal sample reached an average qualitative similarity of only 77.1% was found. Therefore, in order to compare the categories in a more adequate manner, a commensurate abundance threshold was implemented. It was found that when applying a cut-off value of 0.19% at least 99% similarity was reached when comparing sequencing sets representing the same fecal sample. However, setting a threshold in a way that all numbers being below a given value would be excised (turned into zero) raised a problem of introducing false dissimilarities between the groups. Therefore a script was applied that whenever two categories shared a given taxon no matter its relative value, this label was considered as shared. The calculated threshold value was further implemented for all intra-groups comparisons.

In the present study the Firmicutes fraction was more abundant than the Bacterioidetes in most fecal and caecal samples, except for the BALB/c fecal samples, which showed an inverted proportion of these two phyla. Human fecal samples had relatively more Firmicutes and less Bacteriodetes compared to most mouse fecal and caecal samples. This is in accordance with earlier studies reporting that the majority of bacteria in the gut are members of these two phyla and that in mice the Firmicutes fraction seems to be much larger than the Bacteriodetes fraction [9], [43]–[47]. Representatives of the TM7 phylum were clearly reduced in human samples (0.001%) compared to mouse fecal (0.1%) and caecal (0.8%) specimens. This is in correspondence with a study by Rawls et al. where human colonic samples were shown to be free of the TM7 phylum in comparison to the mouse cecum and zebrafish gut microbiota. However, a relatively low number of sequences was used (less than 3000) which would not be enough to detect representatives of this phylum at the similar abundance levels [48].

As seen from Fig. 6 increased sequencing depth disclose consecutive phylogroups (phyla and genus level) resulting in higher rates of similarity between the two categories, mice and humans with increased sequencing dept. Although the GM classified at the phylum level could be well explored with relatively few sequences [9], information at the genus level and its link with diseases in general require much deeper sequencing as also evident from Fig. 6.

For example, the *Prevotella* genus has been found to be inversely correlated with body weight gain, cholesterol accumulation, insulin resistance and diet-induced adiposity [49] and this genus was exclusive for the BALB/c fecal and caecal microbiota but not detected in any of the remaining mouse strains. In addition, the fecal microbiota of the NOD mice was the only one lacking representatives of the *Odoribacter* genus which relative abundance was recently shown to be increased in the caecum of mice exposed to grid floor induced stress [14]. Unclassified members from the Proteobacteria phylum were unique for the fecal and caecal samples of BALB/c mice and adult B6.V-*Lep^ob^*/J group that on the other hand were lacking members of the *Akkermansia* genus. *Akkermansia muciniphila* has been suggested to possess anti-inflammatory properties as it was found to be present in lower levels in humans suffering from inflammatory bowel disease compare to the healthy control group [50] and greatly increased in vancomycin treated NOD whose cumulative diabetes incidence was significantly reduced [6]. A single unclassified genus was found to be the only qualitative difference between the fecal and caecal content of the two BALB/c groups with no differences at the phyla level and no major divergence in the species richness or diversity. It could be thus concluded that the main cause of differences between samples representing GM pictures of two parts of the BALB/c mice gastrointestinal track was the rearrangement in the bacterial relative abundance. It has previously been shown using the Denaturation Gradient Gel Electrophoresis (DGGE) that profiles of fecal and caecal microbiota do not cluster in the same way proving that the GM of an individual presents different proportions in species abundance along the GI track [51].

Pairwise comparison (mice *vs.* humans) using 0.6 million reads per category disclosed 89% similarity between mouse and human GM genera, with 9 genera being unique for human samples and not detected in any of the three mice strains (abundance threshold 0.19%). Among these 9 genera were *Faecalibacterium* from the Ruminococcaeae family and *Asteroleplasma* from phylum Tenericutes that both have been suggested as possible indicators of a healthy human GM since disturbances in the relative distribution of common species from these genera have been linked with the etiology of Crohn’s disease (CD) and ulcerative colitis (UC) [52]–[55]. The *Megasphera* genus was also unique for humans not being detected in any of the three mouse strains. *Megasphera* spp. have been imputed to support the growth of colonic mucosa [56], [57]. The *Mistsuokella* genus has recently been identified as a GM member of lean as well as obese Indians [58] Many bacterial communities from the Clostridia class including genera that were found unique for humans such as *Faecalibacterium* and *Dialister* but also *Sutterella* from the Proteobacteria phylum showed poor establishment after transplanting them from human into mice GI tracks [59]. It seems possible that mouse genetics disfavors certain groups of bacteria as germ-free rats presented higher recovery rate of these subgroups [59].

A few of the genera found to be unique for humans in this study have in earlier studies been isolated from the gut of other mice strains or mice from other vendors [60]. Werner et al. reported that among others the *Succinivibrio* and *Faecalibacterium* genera were present in the wild type and TNF ^ΔARE/WT^ mice [61] and Sutterella constituted up to 1% of in the GM in the lean control group of C57BL/J6 mice obtained from Harlan (Oxon, UK). The phylum Deferribacteres was unique in mice compared to the human samples. The only genus representing this phylum is *Mucispirillum* a spiral-shaped bacteria previously found to colonize the mucus layer of the GI tract of laboratory mice [62].

To our knowledge the present study is the broadest comparison of GM consortia between humans and laboratory mice showing that despite immense differences in the bacterial relative abundance both mouse fecal, caecal and human fecal samples share to a large extent, not only representatives of the same phyla, but also a substantial fraction of common genera, which vindicates mice as a human experimental model.

## Supporting Information

Figure S1 **Shared and group-unique genera.** The network presenting shared taxonomic GM groups between human (red node) and mouse (blue node) categories generated for normalized OTU tables (600000 reads per category) after filtering the low abundant OTUs (abundance threshold for unshared taxa = 0,19%) using the make_otu_network.py script (QIIME). The visualization of the OTU-networks was performed with an open source platform –Cytoscape (version 2.8.3, http://www.cytoscape.org/).
(PDF)

Figure S2 **Number of GM phyla and genera shared between given categories using raw data.** Number of taxonomic labels shared between given mice strains using raw data where no abundance threshold was used **(A)** on the genus level and **(B)** on the phylum level. 80000 high quality 16s rRNA reads used to represent the GM of each mouse strain were annotated to the Ribosomal Database Project (RDP, http://rdp.cme.msu.edu/) database. **(C)** Number of phyla and genera shared between collated categories of humans and mice using raw data (600000 reads per category). Labels “BALB/c _(f)_”, “BALB/c _(c)_”, “B6.V-*Lep^ob^*/J _(16)_” and “B6.V-*Lep^ob^*/J _(8)_” stand for the gut GM of BALB/c mice determined using fecal and caecal samples and B6.V-*Lep^ob^*/J mice using fecal specimens sampled in 16 and 8 weeks of age respectively.
(PDF)

Table S1 **The relative distribution of bacterial genera among categories.** The relative distribution of the gut microbial genera among single human (16 individuals) and five mouse groups (15, 14, 19, 19, 21 mice respectively). The abundance threshold within at least one of the category was set to 0.19%.
(PDF)

Table S2 **Differences in the relative abundance of gut microbial phyla between categories.** Differences in the gut microbial phyla relative distribution verified using Metastats (http://metastats.cbcb.umd.edu) for all combinations of categories. Each pairwise comparison was performed based on 1000 permutations (p value threshold = 0.05, q value threshold = 0.5).
(PDF)

Table S3 **Differences in the relative abundance of gut microbial genera between categories.** Differences in the gut microbial genera relative distribution verified using Metastats (http://metastats.cbcb.umd.edu) for all combinations of categories. Each pairwise comparison was performed based on 1000 permutations (p value threshold = 0.05, q value threshold = 0.5).
(PDF)
