# Supplementary figures and images for: Quantitatively Different, yet Qualitatively Alike: A Meta-Analysis of the Mouse Core Gut Microbiome with a View towards the Human Gut Microbiome
Source: PLoS One. 2013 May 1;8(5):e62578. doi: 10.1371/journal.pone.0062578 (PMC3641060; doi:10.1371/journal.pone.0062578)

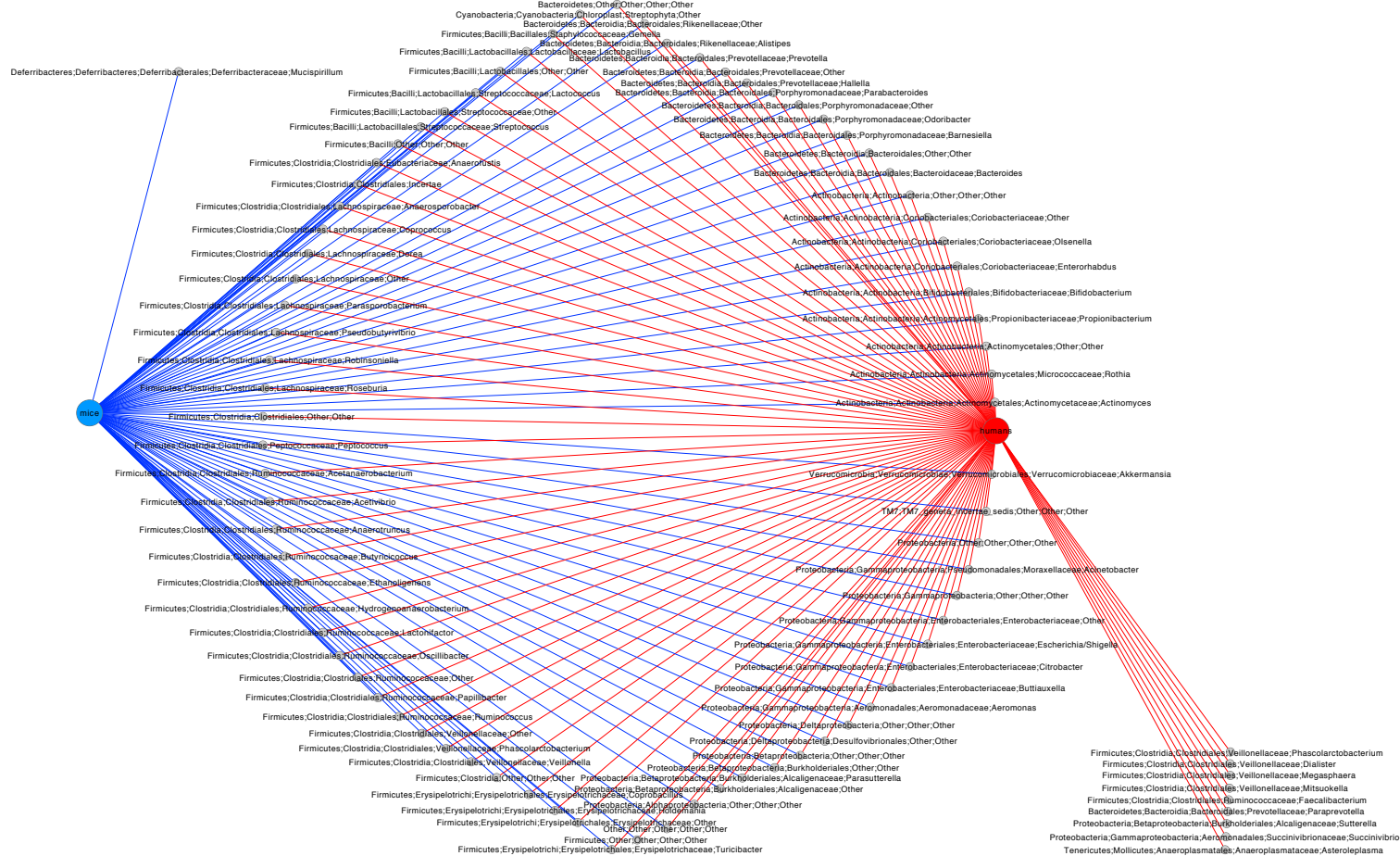

Supplement: Figure S1 — Shared and group-unique genera. The network presenting shared taxonomic GM groups between human (red node) and mouse (blue node) categories generated for normalized OTU tables (600000 reads per category) after filtering the low abundant OTUs (abundance threshold for unshared taxa = 0,19%) using the make_otu_network.py script (QIIME). The visualization of the OTU-networks was performed with an open source platform –Cytoscape (version 2.8.3, http://www.cytoscape.org/). (PDF) [file pone.0062578.s001.pdf]
