# Supplementary material for: Quantitatively Different, yet Qualitatively Alike: A Meta-Analysis of the Mouse Core Gut Microbiome with a View towards the Human Gut Microbiome
Source: PLoS One. 2013 May 1;8(5):e62578. doi: 10.1371/journal.pone.0062578 (PMC3641060; doi:10.1371/journal.pone.0062578)
Supplement: Figure S2 — Number of GM phyla and genera shared between given categories using raw data. Number of taxonomic labels shared between given mice strains using raw data where no abundance threshold was used (A) on the genus level and (B) on the phylum level. 80000 high quality 16s rRNA reads used to represent the GM of each mouse strain were annotated to the Ribosomal Database Project (RDP, http://rdp.cme.msu.edu/) database. (C) Number of phyla and genera shared between collated categories of humans and mice using raw data (600000 reads per category). Labels “BALB/c (f)”, “BALB/c (c)”, “B6.V-Lepob/J (16)” and “B6.V-Lepob/J (8)” stand for the gut GM of BALB/c mice determined using fecal and caecal samples and B6.V-Lepob/J mice using fecal specimens sampled in 16 and 8 weeks of age respectively. (PDF) [file pone.0062578.s002.pdf]

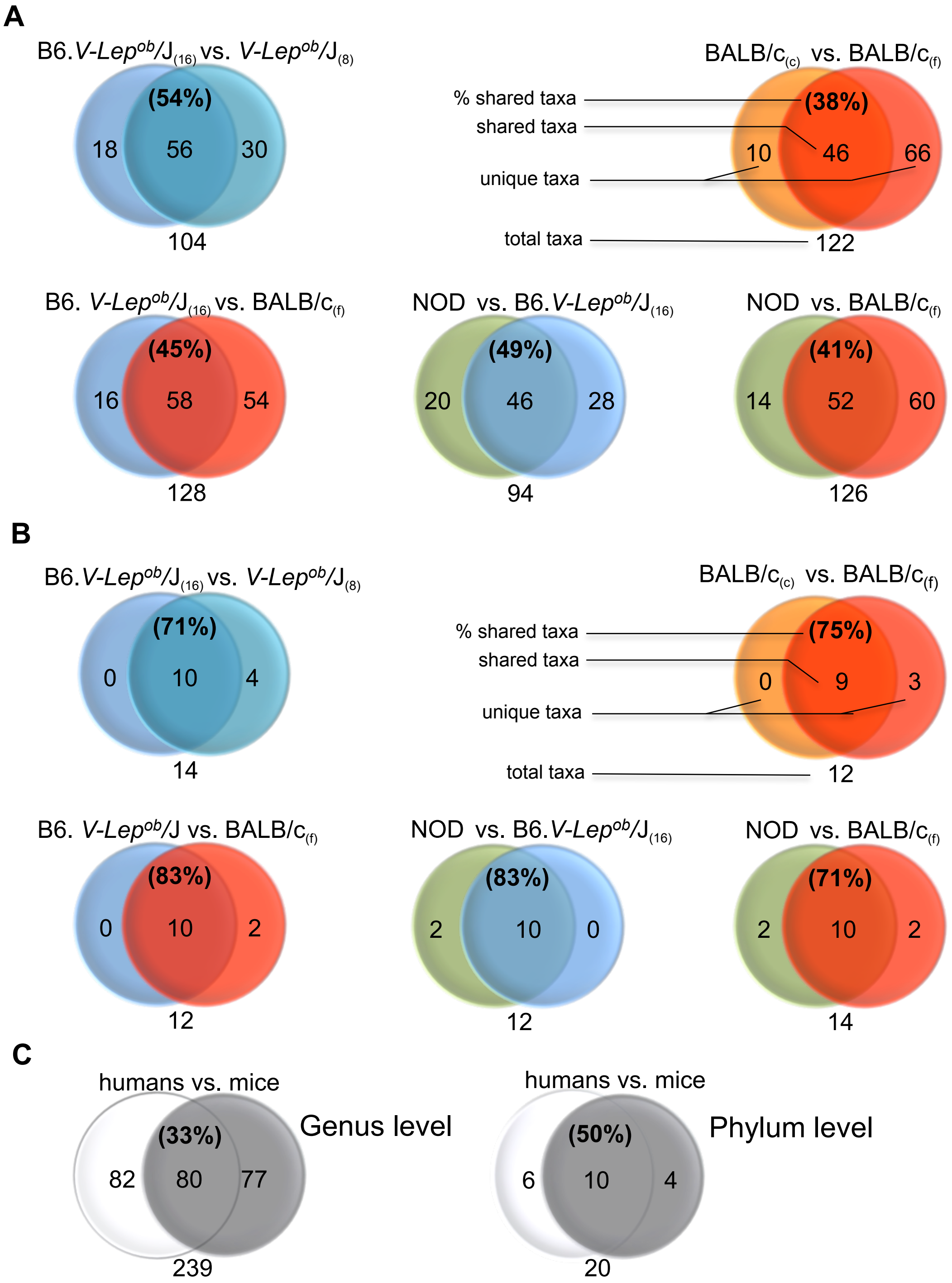
A
B6.V-Lep^ob/J(16) vs. V-Lep^ob/J(8)
(54%)
18
56
30
104
BALB/c(c) vs. BALB/c(f)
% shared taxa
shared taxa
unique taxa
total taxa
(38%)
10
46
66
122
B6. V-Lep^ob/J(16) vs. BALB/c(f)
(45%)
16
58
54
128
NOD vs. B6.V-Lep^ob/J(16)
(49%)
20
46
28
94
NOD vs. BALB/c(f)
(41%)
14
52
60
126
B
B6.V-Lep^ob/J(16) vs. V-Lep^ob/J(8)
(71%)
0
10
4
14
BALB/c(c) vs. BALB/c(f)
% shared taxa
shared taxa
unique taxa
total taxa
(75%)
0
9
3
12
B6. V-Lep^ob/J vs. BALB/c(f)
(83%)
0
10
2
12
NOD vs. B6.V-Lep^ob/J(16)
(83%)
2
10
0
12
NOD vs. BALB/c(f)
(71%)
2
10
2
14
C
humans vs. mice
Genus level
(33%)
82
80
77
239
humans vs. mice
Phylum level
(50%)
6
10
4
20
