# Supplementary material for: Quantitatively Different, yet Qualitatively Alike: A Meta-Analysis of the Mouse Core Gut Microbiome with a View towards the Human Gut Microbiome
Source: PLoS One. 2013 May 1;8(5):e62578. doi: 10.1371/journal.pone.0062578 (PMC3641060; doi:10.1371/journal.pone.0062578)
Supplement: Table S2 — Differences in the relative abundance of gut microbial phyla between categories. Differences in the gut microbial phyla relative distribution verified using Metastats (http://metastats.cbcb.umd.edu) for all combinations of categories. Each pairwise comparison was performed based on 1000 permutations (p value threshold = 0.05, q value threshold = 0.5). (PDF) [file pone.0062578.s004.pdf]

Table S2. Differences in the relative abundance of gut microbial phyla between categories.

phyla	BALB(caecum)/ B6.V-Lep^{ob}/J (8 weeks)		BALB(caecum)/ BALB(feces)		B6.V-Lep^{ob}/J (8weeks) /B6.V-Lep^{ob}/J (16weeks)		B6.V-Lep^{ob}/J (16weeks)/ BALB(feces)		B6.V-Lep^{ob}/J (8weeks)/ BALB(feces)		NOD/ B6.V-Lep^{ob}/J (16weeks)		NOD/ B6.V-Lep^{ob}/J (8weeks)		NOD/ BALB (caecum)		NOD/ BALB(feces)		BALB(caecum)/ B6.V-Lep^{ob}/J (16 weeks)		BALB(caecum)/ human		B6.V-Lep^{ob}/J (16weeks)/ human		B6.V-Lep^{ob}/J (8weeks)/ human		NOD/ human		BALB(feces)/ human	
	p	q	p	q	p	q	p	q	p	q	p	q	p	q	p	q	p	q	p	q	p	q	p	q	p	q	p	q	p	q
**Actinobacteria**	-	-	0.012	0.245	-	-	-	-	0.001	0.045	-	-	-	-	-	-	-	-	-	-	0.001	0.018	0.001	0.009	0.001	0.008	0.009	0.122	0.001	0.008
**Bacteroidetes**	0.001	0.013	0.001	0.041	-	-	0.001	0.044	-	-	-	-	0.002	0.086	-	-	0.001	0.025	0.002	0.05	0.03	0.264	0.001	0.009	0.001	0.008	-	-	0.001	0.008
**Deferribacteres**	-	-	0.001	0.041	-	-	0.001	0.013	-	-	-	-	-	-	-	-	0.011	0.193	0.001	0.042	0.001	0.018	0.001	0.009	0.001	0.008	0.001	0.027	0.001	0.008
**Firmicutes**	0.001	0.013	0.001	0.041	-	-	0.001	0.044	-	-	-	-	-	-	-	-	0.002	0.041	-	-	-	-	0.015	0.09	0.001	0.008	-	-	0.001	0.008
**Unclassified phylum**	-	-	-	-	-	-	-	-	-	-	-	-	-	-	-	-	-	-	0.003	0.063	0.007	0.07	-	-	0.049	0.215	-	-	-	-
**Proteobacteria**	0.001	0.013	-	-	-	-	0.001	0.044	0.007	0.127	0.001	0.035	0.001	0.086	0.001	0.041	0.001	0.025	0.001	0.042	-	-	0.001	0.009	0.001	0.008	0.001	0.027	0.048	0.343
**TM7**	0.001	0.013	0.004	0.098	-	-	0.011	0.367	0.019	0.287	0.001	0.035	0.007	0.15	0.001	0.041	0.001	0.025	0.001	0.042	0.001	0.018	0.001	0.009	0.001	0.008	0.04	0.466	0.001	0.008
**Tenericutes**	0.029	0.295	-	-	-	-	-	-	0.003	0.068	0.001	0.035	-	-	0.002	0.062	0.001	0.025	-	-	-	-	-	-	-	-	-	-	-	-
**Verrucomicrobia**	0.001	0.013	-	-	0.001	0.129	-	-	0.001	0.045	0.001	0.035	-	-	0.001	0.041	0.001	0.025	-	-	0.001	0.018	0.001	0.009	-	-	-	-	0.001	0.008

Differences in the gut microbial phyla relative distribution verified using Metastats (http://metastats.cbcb.umd.edu) for all combinations of categories. Each pairwise comparison was performed based on 1000 permutations (p value threshold =0.05, q value threshold =0.5).
