# Supplementary material for: Quantitatively Different, yet Qualitatively Alike: A Meta-Analysis of the Mouse Core Gut Microbiome with a View towards the Human Gut Microbiome
Source: PLoS One. 2013 May 1;8(5):e62578. doi: 10.1371/journal.pone.0062578 (PMC3641060; doi:10.1371/journal.pone.0062578)
Supplement: Table S3 — Differences in the relative abundance of gut microbial genera between categories. Differences in the gut microbial genera relative distribution verified using Metastats (http://metastats.cbcb.umd.edu) for all combinations of categories. Each pairwise comparison was performed based on 1000 permutations (p value threshold = 0.05, q value threshold = 0.5). (PDF) [file pone.0062578.s005.pdf]

Table S3. Differences in the relative abundance of gut microbial genera between categories.

Phylum;Class;Order;Family;Genus	B6(8 weeks)/B6(16 weeks)		NOD/BALB (caecum)		NOD/B6 (16weeks)		NOD/B6 (8weeks)		NOD/BALB(feces)		BALB(caecum)/B6(16 weeks)		BALB(caecum)/B6(8 weeks)		BALB(caecum)/BALB(feces)		B6(16 weeks)/BALB(feces)		B6(8 weeks)/BALB(feces)		B6(8 weeks)/human		BALB(feces)/human		B6(16 weeks)/human		NOD/human		BALB(caecum)/human	
	p	q	p	q	p	q	p	q	p	q	p	q	p	q	p	q	p	q	p	q	p	q	p	q	p	q	p	q	p	q
Actinobacteria;Actinobacteria;Actinomycetales;Pseudonocardiaceae;Pseudonocardia	-	-	-	-	-	-	-	-	-	-	-	-	-	-	-	-	0.005	0.292	-	-	-	-	-	-	-	-	-	-	-	-
Actinobacteria;Actinobacteria;Bifidobacteriales;Bifidobacteriaceae;Bifidobacterium	-	-	-	-	-	-	-	-	-	-	-	-	-	-	-	-	-	-	-	-	0.001	0.001	0.001	0.001	0.001	0.001	0.001	0.000	0.001	0.000
Actinobacteria;Actinobacteria;Coriobacteriales;Coriobacteriaceae;Enterorhabdus	-	-	-	-	-	-	-	-	-	-	-	-	-	-	-	-	-	-	-	-	-	-	-	-	-	-	-	-	-	-
Actinobacteria;Actinobacteria;Coriobacteriales;Coriobacteriaceae;Olsenella	-	-	-	-	-	-	-	-	-	-	-	-	-	-	-	-	0.001	0.107	-	-	-	-	-	-	-	-	-	-	-	-
Bacteroidetes;Bacteroidia;Bacteroidales;Bacteroidaceae;Bacteroides	0.001	0.018	0.001	0.000	0.034	0.040	-	-	-	-	0.030	0.021	0.001	0.000	0.001	0.000	0.001	0.107	-	-	0.003	0.003	0.001	0.001	0.001	0.001	0.001	0.000	0.001	0.000
Bacteroidetes;Bacteroidia;Bacteroidales;Other;Other	-	-	-	-	0.001	0.002	-	-	0.001	0.001	0.015	0.012	-	-	0.001	0.000	0.002	0.151	0.001	0.004	-	-	0.001	0.001	-	-	0.001	0.000	-	-
Bacteroidetes;Bacteroidia;Bacteroidales;Porphyromonadaceae;Barnesiella	-	-	0.035	0.000	0.014	0.021	-	-	-	-	0.001	0.001	0.001	0.000	0.003	0.000	0.002	0.151	0.002	0.006	0.001	0.001	0.001	0.001	0.001	0.001	0.002	0.000	0.026	0.008
Bacteroidetes;Bacteroidia;Bacteroidales;Porphyromonadaceae;Odoribacter	0.016	0.093	0.001	0.000	0.001	0.002	0.001	0.000	0.001	0.001	0.001	0.001	0.001	0.000	-	-	0.005	0.292	0.001	0.004	0.001	0.001	0.001	0.001	0.001	0.001	-	-	0.001	0.000
Bacteroidetes;Bacteroidia;Bacteroidales;Porphyromonadaceae;Other	-	-	-	-	-	-	-	-	-	-	-	-	-	-	0.005	0.000	-	-	0.009	0.027	0.001	0.001	0.001	0.001	0.001	0.001	0.001	0.000	0.001	0.000
Bacteroidetes;Bacteroidia;Bacteroidales;Porphyromonadaceae;Parabacteroides	-	-	-	-	-	-	-	-	-	-	-	-	-	-	-	-	0.001	0.107	-	-	0.001	0.001	0.039	0.023	0.001	0.001	0.001	0.000	0.001	0.000
Bacteroidetes;Bacteroidia;Bacteroidales;Prevotellaceae;Other	-	-	-	-	0.013	0.021	0.019	0.000	0.006	0.007	0.001	0.001	0.001	0.000	0.002	0.000	-	-	-	-	-	-	-	-	-	-	-	-	-	-
Bacteroidetes;Bacteroidia;Bacteroidales;Prevotellaceae;Prevotella	-	-	-	-	-	-	-	-	0.001	0.001	-	-	-	-	0.004	0.000	0.002	0.151	0.001	0.004	0.001	0.001	-	-	0.001	0.001	0.001	0.000	0.001	0.000
Bacteroidetes;Bacteroidia;Bacteroidales;Rikenellaceae;Alistipes	0.002	0.018	0.002	0.000	0.001	0.002	0.001	0.000	0.001	0.001	-	-	0.001	0.000	0.001	0.000	0.001	0.107	0.001	0.004	0.001	0.001	0.001	0.001	0.001	0.001	0.004	0.000	0.001	0.000
Bacteroidetes;Bacteroidia;Bacteroidales;Rikenellaceae;Other	-	-	-	-	-	-	-	-	-	-	-	-	-	-	-	-	0.001	0.107	-	-	0.001	0.001	-	-	0.001	0.001	0.001	0.000	0.009	0.003
Bacteroidetes;Other;Other;Other;Other	-	-	0.002	0.000	0.001	0.002	0.001	0.000	0.007	0.008	-	-	-	-	-	-	-	-	-	-	-	-	-	-	-	-	-	-	-	-
Deferribacteres;Deferribacteres;Deferribacterales;Deferribacteraceae;Mucispirillum	-	-	0.045	0.000	0.030	0.040	0.004	0.000	0.014	0.013	0.001	0.001	0.001	0.000	0.001	0.000	-	-	-	-	0.001	0.001	-	-	-	-	0.001	0.000	0.001	0.000
Firmicutes;Bacilli;Lactobacillales;Lactobacillaceae;Lactobacillus	-	-	-	-	-	-	-	-	-	-	-	-	-	-	0.001	0.000	0.001	0.107	0.001	0.004	-	-	0.001	0.001	-	-	0.009	0.000	-	-
Firmicutes;Bacilli;Lactobacillales;Streptococcaceae;Streptococcus	-	-	-	-	-	-	-	-	-	-	-	-	-	-	-	-	-	-	-	-	0.001	0.001	0.001	0.001	0.001	0.001	-	-	-	-
Firmicutes;Clostridia;Clostridiales;Incertae Sedis XIV;Blautia	-	-	-	-	-	-	-	-	-	-	-	-	-	-	-	-	0.001	0.126	-	-	0.001	0.001	0.001	0.001	0.001	0.001	0.001	0.000	0.001	0.000
Firmicutes;Clostridia;Clostridiales;Lachnospiraceae;Coprococcus	-	-	-	-	-	-	-	-	-	-	-	-	-	-	-	-	-	-	-	-	0.001	0.001	0.001	0.001	0.001	0.001	0.001	0.000	0.001	0.000
Firmicutes;Clostridia;Clostridiales;Lachnospiraceae;Dorea	-	-	-	-	0.001	0.002	0.001	0.000	-	-	0.001	0.001	0.001	0.000	-	-	0.002	0.151	0.001	0.004	-	-	0.001	0.001	-	-	0.001	0.000	0.001	0.000
Firmicutes;Clostridia;Clostridiales;Lachnospiraceae;Other	0.018	0.093	-	-	-	-	-	-	0.001	0.001	-	-	-	-	0.001	0.000	0.001	0.107	0.002	0.006	0.001	0.001	0.020	0.012	0.001	0.001	0.001	0.000	0.001	0.000
Firmicutes;Clostridia;Clostridiales;Lachnospiraceae;Roseburia	-	-	-	-	-	-	-	-	-	-	0.001	0.001	0.001	0.000	-	-	0.001	0.107	0.021	0.050	0.001	0.001	0.001	0.001	0.001	0.001	0.001	0.000	0.001	0.000
Firmicutes;Clostridia;Clostridiales;Other;Other	-	-	0.001	0.000	-	-	0.004	0.000	0.011	0.011	0.001	0.001	0.001	0.000	-	-	-	-	0.001	0.004	-	-	-	-	-	-	-	-	0.035	0.010
Firmicutes;Clostridia;Clostridiales;Peptococcaceae;Other	-	-	-	-	-	-	-	-	-	-	-	-	-	-	-	-	0.004	0.281	-	-	-	-	-	-	-	-	-	-	-	-
Firmicutes;Clostridia;Clostridiales;Ruminococcaceae;Anaerotruncus	0.002	0.018	-	-	0.034	0.040	-	-	-	-	0.004	0.003	-	-	-	-	0.004	0.281	-	-	0.001	0.001	0.001	0.001	0.001	0.001	0.001	0.000	0.001	0.000
Firmicutes;Clostridia;Clostridiales;Ruminococcaceae;Butyricicoccus	-	-	-	-	-	-	-	-	-	-	-	-	0.019	0.000	0.041	0.000	-	-	-	-	-	-	-	-	-	-	-	-	-	-
Firmicutes;Clostridia;Clostridiales;Ruminococcaceae;Faecalibacterium	-	-	-	-	-	-	-	-	-	-	-	-	-	-	-	-	-	-	-	-	0.001	0.001	0.001	0.001	0.001	0.001	0.001	0.000	0.001	0.000
Firmicutes;Clostridia;Clostridiales;Ruminococcaceae;Oscillibacter	0.037	0.167	0.001	0.000	-	-	-	-	-	-	0.001	0.001	0.006	0.000	0.025	0.000	-	-	-	-	-	-	-	-	-	-	-	-	-	-
Firmicutes;Clostridia;Clostridiales;Ruminococcaceae;Other	-	-	0.001	0.000	-	-	-	-	-	-	0.001	0.001	0.002	0.000	0.001	0.000	-	-	-	-	-	-	-	-	-	-	-	-	-	-
Firmicutes;Clostridia;Clostridiales;Ruminococcaceae;Papillibacter	-	-	-	-	-	-	-	-	-	-	0.019	0.014	0.001	0.000	0.030	0.000	-	-	0.018	0.046	-	-	0.001	0.001	0.001	0.001	0.001	0.000	0.001	0.000
Firmicutes;Clostridia;Clostridiales;Ruminococcaceae;Ruminococcus	-	-	-	-	-	-	-	-	-	-	-	-	-	-	-	-	-	-	-	-	0.001	0.001	0.013	0.008	0.001	0.001	0.001	0.000	0.001	0.000
Firmicutes;Clostridia;Clostridiales;Ruminococcaceae;Subdoligranulum	-	-	-	-	-	-	-	-	-	-	-	-	-	-	-	-	-	-	-	-	0.001	0.001	0.001	0.001	0.001	0.001	0.001	0.000	0.001	0.000
Firmicutes;Clostridia;Clostridiales;Veillonellaceae;Dialister	-	-	-	-	-	-	-	-	-	-	-	-	-	-	-	-	-	-	-	-	0.001	0.001	0.001	0.001	0.001	0.001	0.001	0.000	0.001	0.000
Firmicutes;Clostridia;Clostridiales;Veillonellaceae;Megasphaera	-	-	-	-	-	-	-	-	-	-	-	-	-	-	-	-	-	-	-	-	0.001	0.001	0.001	0.001	0.001	0.001	0.001	0.000	-	-
Firmicutes;Clostridia;Clostridiales;Veillonellaceae;Mitsuokella	-	-	-	-	-	-	-	-	-	-	-	-	-	-	-	-	-	-	-	-	0.001	0.001	0.001	0.001	0.001	0.001	0.001	0.000	0.001	0.000
Firmicutes;Clostridia;Clostridiales;Veillonellaceae;Phascolarctobacterium	-	-	-	-	-	-	-	-	-	-	-	-	-	-	-	-	-	-	-	-	0.001	0.001	0.001	0.001	0.001	0.001	0.001	0.000	0.001	0.000
Firmicutes;Clostridia;Other;Other;Other	-	-	-	-	0.001	0.002	0.001	0.000	0.003	0.004	0.001	0.001	0.001	0.000	0.038	0.000	-	-	-	-	0.001	0.001	0.022	0.013	0.001	0.001	-	-	-	-
Firmicutes;Erysipelotrichi;Erysipelotrichales;Erysipelotrichaceae;Other	-	-	-	-	-	-	-	-	-	-	-	-	-	-	-	-	0.005	0.292	-	-	0.004	0.004	-	-	0.001	0.001	0.004	0.000	0.001	0.000
Firmicutes;Other;Other;Other;Other	-	-	-	-	-	-	-	-	-	-	-	-	-	-	-	-	-	-	0.040	0.091	0.005	0.005	-	-	0.031	0.030	0.043	0.000	0.011	0.004
Proteobacteria;Alphaproteobacteria;Other;Other;Other	-	-	-	-	-	-	-	-	-	-	-	-	-	-	-	-	0.006	0.335	-	-	-	-	0.001	0.001	-	-	0.001	0.000	0.001	0.000
Proteobacteria;Betaproteobacteria;Burkholderiales;Alcaligenaceae;Other	-	-	-	-	-	-	-	-	-	-	-	-	-	-	-	-	-	-	-	-	-	-	-	-	-	-	-	-	-	-
Proteobacteria;Betaproteobacteria;Burkholderiales;Alcaligenaceae;Parasutterella	-	-	-	-	-	-	-	-	-	-	-	-	-	-	-	-	0.001	0.107	-	-	0.001	0.001	0.008	0.005	0.001	0.001	0.015	0.000	0.002	0.001
Proteobacteria;Deltaproteobacteria;Desulfovibrionales;Desulfovibrionaceae;Desulfovibrio	-	-	-	-	-	-	-	-	-	-	-	-	-	-	-	-	-	-	-	-	-	-	-	-	-	-	0.001	0.000	0.001	0.000
Proteobacteria;Deltaproteobacteria;Desulfovibrionales;Desulfovibrionaceae;Other	-	-	-	-	-	-	-	-	-	-	-	-	-	-	-	-	-	-	-	-	0.001	0.001	0.001	0.001	0.001	0.001	0.001	0.000	0.001	0.000
Proteobacteria;Deltaproteobacteria;Desulfovibrionales;Other;Other	-	-	0.001	0.000	-	-	0.001	0.000	-	-	-	-	-	-	0.001	0.000	0.001	0.107	-	-	0.001	0.001	-	-	-	-	-	-	0.001	0.000
Proteobacteria;Deltaproteobacteria;Other;Other;Other	-	-	-	-	-	-	-	-	0.001	0.001	-	-	-	-	-	-	0.001	0.107	0.001	0.004	-	-	0.001	0.001	-	-	-	-	-	-
Proteobacteria;Gammaproteobacteria;Aeromonadales;Succinivibrionaceae;Succinivibrio	-	-	-	-	-	-	-	-	-	-	-	-	-	-	-	-	-	-	-	-	0.001	0.001	0.001	0.001	0.001	0.001	0.001	0.000	0.001	0.000
Proteobacteria;Gammaproteobacteria;Enterobacteriales;Enterobacteriaceae;Citrobacter	-	-	-	-	-	-	-	-	-	-	-	-	-	-	-	-	-	-	-	-	-	-	-	-	-	-	-	-	-	-
Proteobacteria;Other;Other;Other;Other	-	-	0.001	0.000	-	-	-	-	0.001	0.001	0.001	0.001	0.001	0.000	-	-	0.001	0.107	0.001	0.004	-	-	0.001	0.001	-	-	-	-	0.001	0.000
TM7;TM7_genera_incertae_sedis;Other;Other;Other	-	-	0.001	0.000	-	-	-	-	0.001	0.001	0.001	0.001	0.001	0.000	0.003	0.000	-	-	0.016	0.044	-	-	0.001	0.001	-	-	-	-	0.001	0.000
Tenericutes;Mollicutes;Anaeroplasmatales;Anaeroplasmataceae;Anaeroplasma	-	-	0.001	0.000	0.001	0.002	-	-	0.001	0.001	-	-	-	-	-	-	-	-	-	-	-	-	-	-	-	-	0.001	0.000	-	-
Tenericutes;Mollicutes;Anaeroplasmatales;Anaeroplasmataceae;Asteroleplasma	-	-	-	-	-	-	-	-	-	-	-	-	-	-	-	-	-	-	-	-	0.001	0.001	0.001	0.001	0.001	0.001	0.001	0.000	0.001	0.000
Verrucomicrobia;Verrucomicrobiae;Verrucomicrobiales;Verrucomicrobiaceae;Akkermansia	0.001	0.018	0.001	0.000	0.001	0.002	-	-	0.001	0.001	-	-	0.001	0.000	-	-	-	-	0.001	0.004	-	-	0.001	0.001	0.001	0.001	-	-	0.001	0.000
Unclassified	0.015	0.093	0.036	0.000	-	-	-	-	-	-	0.003	0.003	-	-	-	-	-	-	-	-	-	-	-	-	-	-	-	-	0.022	0.007

Differences in the gut microbial genera relative distribution verified using Metastats (http://metastats.cbcb.umd.edu) for all combinations of categories. Each pairwise comparison was performed based on 1000 permutations (p value threshold =0.05, q value threshold =0.5).
