# Supplementary material for: Quantitatively Different, yet Qualitatively Alike: A Meta-Analysis of the Mouse Core Gut Microbiome with a View towards the Human Gut Microbiome
Source: PLoS One. 2013 May 1;8(5):e62578. doi: 10.1371/journal.pone.0062578 (PMC3641060; doi:10.1371/journal.pone.0062578)
Supplement: Table S1 — The relative distribution of bacterial genera among categories. The relative distribution of the gut microbial genera among single human (16 individuals) and five mouse groups (15, 14, 19, 19, 21 mice respectively). The abundance threshold within at least one of the category was set to 0.19%. (PDF) [file pone.0062578.s003.pdf]

Table S1. The relative distribution of genera among categories.

Phulum;Class;Order;Family;Genus	B6. V-Lepob/J (8 weeks)	B6. V-Lepob/J 16(weeks	BALB/c caecum	BALB/c (feces)	NOD	humans
Acidobacteria;Acidobacteria_Gp1;Gp1;Other;Other	-	0.000665655	-	-	-	-
Acidobacteria;Acidobacteria_Gp16;Gp16;Other;Other	-	0.001996965	-	-	-	-
Acidobacteria;Acidobacteria_Gp18;Gp18;Other;Other	-	0.000665655	-	-	-	-
Acidobacteria;Acidobacteria_Gp2;Gp2;Other;Other	-	0.00133131	-	-	-	-
Acidobacteria;Acidobacteria_Gp3;Gp3;Other;Other	0.000701661	0.00133131	-	0.000491193	-	-
Acidobacteria;Acidobacteria_Gp4;Gp4;Other;Other	-	-	-	0.000491193	0.000843775	-
Acidobacteria;Acidobacteria_Gp7;Gp7;Other;Other	-	0.00133131	-	-	-	-
Actinobacteria;Actinobacteria;Acidimicrobiales;Other;Other	-	0.000665655	-	-	-	-
Actinobacteria;Actinobacteria;Acidimicrobidae_incertae_sedis;Ilumatobacter;Other	-	-	-	0.000491193	-	-
Actinobacteria;Actinobacteria;Actinomycetales;Actinomycetaceae;Actinobaculum	-	-	-	0.000491193	-	-
Actinobacteria;Actinobacteria;Actinomycetales;Actinomycetaceae;Actinomyces	0.000701661	-	-	-	-	0.004
Actinobacteria;Actinobacteria;Actinomycetales;Actinomycetaceae;Mobiluncus	-	-	-	-	-	0.000666667
Actinobacteria;Actinobacteria;Actinomycetales;Actinomycetaceae;Varibaculum	-	-	-	-	-	0.000833333
Actinobacteria;Actinobacteria;Actinomycetales;Corynebacteriaceae;Corynebacterium	-	-	-	0.000491193	0.000843775	-
Actinobacteria;Actinobacteria;Actinomycetales;Dermabacteriaceae;Brachybacterium	0.001403322	-	-	-	-	-
Actinobacteria;Actinobacteria;Actinomycetales;Microbacteriaceae;Other	0.000701661	0.00133131	-	0.000491193	-	-
Actinobacteria;Actinobacteria;Actinomycetales;Micrococcaceae;Kocuria	0.000701661	-	-	-	-	-
Actinobacteria;Actinobacteria;Actinomycetales;Micrococcaceae;Rothia	-	-	-	-	0.000843775	0.0085
Actinobacteria;Actinobacteria;Actinomycetales;Mycobacteriaceae;Mycobacterium	-	0.000665655	-	-	-	-
Actinobacteria;Actinobacteria;Actinomycetales;Other;Other	-	0.005990894	-	0.002455965	0.002531325	0.001666667
Actinobacteria;Actinobacteria;Actinomycetales;Propionibacteriaceae;Propionibacterium	-	-	-	0.000491193	-	0.000166667
Actinobacteria;Actinobacteria;Actinomycetales;Pseudonocardiaceae;Actinomycetospora	-	-	-	0.000491193	-	-
Actinobacteria;Actinobacteria;Actinomycetales;Pseudonocardiaceae;Other	-	-	-	0.000982386	-	-
Actinobacteria;Actinobacteria;Actinomycetales;Pseudonocardiaceae;Pseudonocardia	0.003508304	0.001996965	-	-	-	-
Actinobacteria;Actinobacteria;Actinomycetales;Streptomycetaceae;Other	-	-	-	0.000491193	-	-
Actinobacteria;Actinobacteria;Bifidobacteriales;Bifidobacteriaceae;Alloscardovia	-	-	-	-	-	0.000166667
Actinobacteria;Actinobacteria;Bifidobacteriales;Bifidobacteriaceae;Bifidobacterium	-	-	0.00301232	0.000491193	-	0.735166667
Actinobacteria;Actinobacteria;Bifidobacteriales;Bifidobacteriaceae;Other	-	-	-	-	-	0.081
Actinobacteria;Actinobacteria;Coriobacteriales;Coriobacteriaceae;Asaccharobacter	-	-	-	-	-	0.003666667
Actinobacteria;Actinobacteria;Coriobacteriales;Coriobacteriaceae;Atopobium	0.000701661	-	-	-	-	0.0005
Actinobacteria;Actinobacteria;Coriobacteriales;Coriobacteriaceae;Collinsella	-	-	-	-	-	0.075333333
Actinobacteria;Actinobacteria;Coriobacteriales;Coriobacteriaceae;Eggerthella	-	-	-	-	-	0.001666667
Actinobacteria;Actinobacteria;Coriobacteriales;Coriobacteriaceae;Enterorhabdus	0.00841993	0.004659584	0.013053388	0.022594874	0.117284732	0.0095
Actinobacteria;Actinobacteria;Coriobacteriales;Coriobacteriaceae;Gordonibacter	-	-	-	-	-	0.001166667
Actinobacteria;Actinobacteria;Coriobacteriales;Coriobacteriaceae;Olsenella	0.009823252	-	0.002008214	-	-	0.021
Actinobacteria;Actinobacteria;Coriobacteriales;Coriobacteriaceae;Other	0.026663112	0.013313097	0.024098563	0.037330661	0.059064253	0.015166667
Actinobacteria;Actinobacteria;Coriobacteriales;Coriobacteriaceae;Slackia	-	-	-	-	-	0.010166667
Actinobacteria;Actinobacteria;Other;Other;Other	-	0.00133131	-	0.000982386	-	0.0025
Actinobacteria;Actinobacteria;Solirubrobacterales;Other;Other	-	0.00133131	-	-	-	-
Bacteria_incertae_sedis;Ktedonobacteria;Ktedonobacterales;Ktedonobacteraceae;Ktedonobacter	-	0.002662619	-	-	-	-
Bacteroidetes;Bacteroidia;Bacteroidales;Bacteroidaceae;Bacteroides	0.349427094	1.545650611	0.158648874	1.905337302	0.885120027	6.168166667
Bacteroidetes;Bacteroidia;Bacteroidales;Other;Other	0.199271676	0.133130974	0.099406573	0.558977533	0.032063452	0.945833333
Bacteroidetes;Bacteroidia;Bacteroidales;Porphyromonadaceae;Barnesiella	6.949248872	6.834944218	1.98411503	3.82688397	4.067839514	0.811833333
Bacteroidetes;Bacteroidia;Bacteroidales;Porphyromonadaceae;Butyricimonas	-	-	-	-	-	0.073
Bacteroidetes;Bacteroidia;Bacteroidales;Porphyromonadaceae;Odoribacter	1.800461693	4.001917086	0.712915826	0.73236863	-	0.047166667
Bacteroidetes;Bacteroidia;Bacteroidales;Porphyromonadaceae;Other	13.2885495	9.480256877	9.922583388	17.82097001	12.44230088	0.352333333
Bacteroidetes;Bacteroidia;Bacteroidales;Porphyromonadaceae;Parabacteroides	0.002806643	0.014644407	0.023094456	0.125745385	0.024469476	0.766666667
Bacteroidetes;Bacteroidia;Bacteroidales;Porphyromonadaceae;Porphyromonas	-	-	-	-	-	0.0045
Bacteroidetes;Bacteroidia;Bacteroidales;Prevotellaceae;Hallella	0.001403322	0.000665655	-	0.001473579	-	0.006666667
Bacteroidetes;Bacteroidia;Bacteroidales;Prevotellaceae;Other	0.373985223	0.240967063	0.077316223	0.342852652	0.113065857	0.409833333
Bacteroidetes;Bacteroidia;Bacteroidales;Prevotellaceae;Paraprevotella	-	-	-	-	-	0.215666667
Bacteroidetes;Bacteroidia;Bacteroidales;Prevotellaceae;Prevotella	-	-	0.002008214	0.991227295	-	5.565166667
Bacteroidetes;Bacteroidia;Bacteroidales;Rikenellaceae;Alistipes	8.328012405	12.47370663	8.379271219	25.18591652	3.770830697	2.066666667
Bacteroidetes;Bacteroidia;Bacteroidales;Rikenellaceae;Other	0.011928234	0.008653513	0.024098563	0.142937137	0.002531325	0.226333333
Bacteroidetes;Flavobacteria;Flavobacteriales;Flavobacteriaceae;Chryseobacterium	-	-	-	0.000982386	-	-
Bacteroidetes;Flavobacteria;Flavobacteriales;Flavobacteriaceae;Cloacibacterium	-	-	-	-	0.000843775	-
Bacteroidetes;Flavobacteria;Flavobacteriales;Flavobacteriaceae;Flavobacterium	-	-	-	0.000982386	-	-
Bacteroidetes;Flavobacteria;Flavobacteriales;Flavobacteriaceae;Other	-	-	-	0.000491193	-	-
Bacteroidetes;Flavobacteria;Flavobacteriales;Other;Other	-	-	-	0.000491193	-	-
Bacteroidetes;Other;Other;Other;Other	0.815329886	0.67364273	0.698858331	0.863517138	0.189849386	0.8195
Bacteroidetes;Sphingobacteria;Sphingobacteriales;Chitinophagaceae;Sediminibacterium	-	-	-	0.000982386	-	-
Bacteroidetes;Sphingobacteria;Sphingobacteriales;Cyclobacteriaceae;Aquiflexum	-	-	-	0.000982386	-	-
Chloroflexi;Anaerolineae;Anaerolineales;Anaerolineaceae;Other	-	-	-	0.000491193	-	0.000166667
Cyanobacteria;Cyanobacteria;Chloroplast;Other;Other	-	-	-	-	-	0.000166667
Cyanobacteria;Cyanobacteria;Chloroplast;Streptophyta;Other	0.028066433	0.029954469	0.002008214	0.040769012	0.020250601	0.007
Cyanobacteria;Cyanobacteria;Family II;GpIIa;Other	-	-	-	0.000491193	-	-
Deferribacteres;Deferribacteres;Deferribacterales;Deferribacteraceae;Mucispirillum	0.183835138	0.15709455	1.048287496	0.12820135	0.485170654	-
Deinococcus-Thermus;Deinococci;Deinococcales;Deinococcaceae;Deinococcus	-	-	-	0.000491193	-	-
Firmicutes;Bacilli;Bacillales;Bacillaceae;Bacillus	-	-	-	0.000982386	0.00168755	-
Firmicutes;Bacilli;Bacillales;Bacillaceae;Other	-	0.00133131	-	-	-	-
Firmicutes;Bacilli;Bacillales;Listeriaceae;Brochothrix	-	-	-	-	0.000843775	-
Firmicutes;Bacilli;Bacillales;Listeriaceae;Listeria	-	-	-	-	-	0.002333333
Firmicutes;Bacilli;Bacillales;Other;Other	-	-	-	-	-	0.000166667
Firmicutes;Bacilli;Bacillales;Paenibacillaceae;Other	-	-	-	-	0.00168755	-
Firmicutes;Bacilli;Bacillales;Planococcaceae;Planomicrobium	-	-	-	0.001473579	-	-
Firmicutes;Bacilli;Bacillales;Staphylococcaceae;Gemella	-	-	0.001004107	-	0.000843775	0.000833333
Firmicutes;Bacilli;Lactobacillales;Aerococcaceae;Abiotrophia	-	-	-	-	-	0.000333333
Firmicutes;Bacilli;Lactobacillales;Carnobacteriaceae;Carnobacterium	-	-	-	0.000491193	-	-
Firmicutes;Bacilli;Lactobacillales;Carnobacteriaceae;Granulicatella	-	-	-	-	-	0.000166667
Firmicutes;Bacilli;Lactobacillales;Carnobacteriaceae;Marinilactibacillus	-	-	-	0.001473579	-	-
Firmicutes;Bacilli;Lactobacillales;Enterococcaceae;Enterococcus	0.001403322	-	-	0.000982386	-	-
Firmicutes;Bacilli;Lactobacillales;Lactobacillaceae;Lactobacillus	0.116475698	0.079878585	0.038147845	0.400322223	0.975403957	0.007
Firmicutes;Bacilli;Lactobacillales;Lactobacillaceae;Other	-	-	-	-	-	0.000166667
Firmicutes;Bacilli;Lactobacillales;Lactobacillaceae;Pediococcus	-	0.000665655	-	-	-	-
Firmicutes;Bacilli;Lactobacillales;Leuconostocaceae;Leuconostoc	0.000701661	0.001996965	-	0.000982386	0.002531325	-
Firmicutes;Bacilli;Lactobacillales;Leuconostocaceae;Weissella	0.023154807	0.029954469	-	0.012771016	0.00168755	-
Firmicutes;Bacilli;Lactobacillales;Other;Other	0.002806643	-	-	0.004420736	0.000843775	0.004666667
Firmicutes;Bacilli;Lactobacillales;Streptococcaceae;Lactococcus	0.006314947	0.012647443	0.00301232	0.014244594	0.017719276	0.0005
Firmicutes;Bacilli;Lactobacillales;Streptococcaceae;Other	-	-	-	-	-	0.000666667
Firmicutes;Bacilli;Lactobacillales;Streptococcaceae;Streptococcus	0.002104982	0.002662619	0.00301232	0.010806244	0.002531325	0.120833333
Firmicutes;Bacilli;Other;Other;Other	0.000701661	-	-	0.000491193	-	0.0015
Firmicutes;Clostridia;Clostridiales;Clostridiaceae;Anaerobacter	-	-	-	-	-	0.001166667
Firmicutes;Clostridia;Clostridiales;Clostridiaceae;Clostridium	-	0.000665655	-	-	-	0.003833333
Firmicutes;Clostridia;Clostridiales;Clostridiaceae;Other	-	-	-	-	-	0.058
Firmicutes;Clostridia;Clostridiales;Clostridiaceae;Tindallia	-	-	-	0.000491193	-	-
Firmicutes;Clostridia;Clostridiales;Eubacteriaceae;Anaerofustis	-	-	-	0.000491193	0.00168755	0.000333333
Firmicutes;Clostridia;Clostridiales;Eubacteriaceae;Eubacterium	-	-	-	-	-	0.019
Firmicutes;Clostridia;Clostridiales;Incertae Sedis XI;Finegoldia	-	-	-	-	-	0.000833333
Firmicutes;Clostridia;Clostridiales;Incertae Sedis XI;Other	-	-	-	-	-	0.038166667
Firmicutes;Clostridia;Clostridiales;Incertae Sedis XI;Parvimonas	-	-	-	-	-	0.001333333
Firmicutes;Clostridia;Clostridiales;Incertae Sedis XI;Peptoniphilus	-	-	-	-	-	0.002
Firmicutes;Clostridia;Clostridiales;Incertae Sedis XII;Fusibacter	-	-	-	0.000491193	-	-
Firmicutes;Clostridia;Clostridiales;Incertae Sedis XIII;Other	-	-	-	-	-	0.000166667
Firmicutes;Clostridia;Clostridiales;Incertae Sedis XIV;Blautia	-	-	0.002008214	0.008350279	-	0.681166667
Firmicutes;Clostridia;Clostridiales;Incertae Sedis XIV;Howardella	-	-	-	-	-	0.023
Firmicutes;Clostridia;Clostridiales;Lachnospiraceae;Anaerosporobacter	-	0.000665655	-	-	-	0.0005
Firmicutes;Clostridia;Clostridiales;Lachnospiraceae;Anaerostipes	-	-	-	-	-	0.001833333
Firmicutes;Clostridia;Clostridiales;Lachnospiraceae;Catonella	-	-	-	-	-	0.0015
Firmicutes;Clostridia;Clostridiales;Lachnospiraceae;Coprococcus	-	0.000665655	0.011045175	0.002947157	0.000843775	2.699833333
Firmicutes;Clostridia;Clostridiales;Lachnospiraceae;Dorea	0.245581291	0.497244189	0.001004107	0.058943149	-	0.247833333
Firmicutes;Clostridia;Clostridiales;Lachnospiraceae;Hespellia	-	-	-	-	-	0.000166667
Firmicutes;Clostridia;Clostridiales;Lachnospiraceae;Lachnobacterium	-	-	-	-	-	0.000166667
Firmicutes;Clostridia;Clostridiales;Lachnospiraceae;Marvinbryantia	0.006314947	0.001996965	0.010041068	0.006385508	0.00843775	-
Firmicutes;Clostridia;Clostridiales;Lachnospiraceae;Moryella	-	-	-	-	-	0.000166667
Firmicutes;Clostridia;Clostridiales;Lachnospiraceae;Oribacterium	-	-	-	-	-	0.000166667
Firmicutes;Clostridia;Clostridiales;Lachnospiraceae;Other	48.9457546	43.57842746	41.52584089	22.50351203	50.93616842	14.48433333
Firmicutes;Clostridia;Clostridiales;Lachnospiraceae;Parasporobacterium	-	-	-	-	-	0.000666667
Firmicutes;Clostridia;Clostridiales;Lachnospiraceae;Pseudobutyrivibrio	-	-	0.001004107	-	-	0.000166667
Firmicutes;Clostridia;Clostridiales;Lachnospiraceae;Robinsoniella	-	-	-	0.007367894	-	0.015833333
Firmicutes;Clostridia;Clostridiales;Lachnospiraceae;Roseburia	-	0.030620124	0.277133476	0.149813838	0.106315656	11.74833333
Firmicutes;Clostridia;Clostridiales;Lachnospiraceae;Syntrophococcus	-	-	-	0.000491193	-	-
Firmicutes;Clostridia;Clostridiales;Other;Other	6.522639087	5.549564662	13.83558755	10.28544085	7.447158688	9.681333333
Firmicutes;Clostridia;Clostridiales;Peptococcaceae;Other	-	-	-	0.007367894	0.005906425	0.016833333
Firmicutes;Clostridia;Clostridiales;Peptococcaceae;Peptococcus	-	-	-	-	-	0.001333333
Firmicutes;Clostridia;Clostridiales;Peptostreptococcaceae;Other	-	-	-	-	-	0.105666667
Firmicutes;Clostridia;Clostridiales;Peptostreptococcaceae;Peptostreptococcus	-	-	-	-	-	0.000166667
Firmicutes;Clostridia;Clostridiales;Peptostreptococcaceae;Sporacetigenium	-	-	-	-	-	0.002
Firmicutes;Clostridia;Clostridiales;Ruminococcaceae;Acetanaerobacterium	-	-	-	-	0.000843775	0.004166667
Firmicutes;Clostridia;Clostridiales;Ruminococcaceae;Acetivibrio	0.003508304	0.001996965	0.005020534	0.002947157	0.010125301	0.010666667
Firmicutes;Clostridia;Clostridiales;Ruminococcaceae;Anaerofilum	-	-	-	-	-	0.005333333
Firmicutes;Clostridia;Clostridiales;Ruminococcaceae;Anaerotruncus	0.184536799	0.412040365	0.395618078	0.305821991	0.547610007	0.014
Firmicutes;Clostridia;Clostridiales;Ruminococcaceae;Butyricicoccus	0.12629895	0.074553346	0.35746202	0.084485181	0.158629709	0.137833333
Firmicutes;Clostridia;Clostridiales;Ruminococcaceae;Ethanoligenens	-	-	-	-	-	0.006166667
Firmicutes;Clostridia;Clostridiales;Ruminococcaceae;Faecalibacterium	-	-	-	-	-	4.274666667
Firmicutes;Clostridia;Clostridiales;Ruminococcaceae;Fastidiosipila	-	-	-	-	-	0.000166667
Firmicutes;Clostridia;Clostridiales;Ruminococcaceae;Hydrogenoanaerobacterium	0.000701661	-	0.006024641	0.000982386	-	0.006333333
Firmicutes;Clostridia;Clostridiales;Ruminococcaceae;Lactonifactor	-	-	-	0.000982386	-	0.002833333
Firmicutes;Clostridia;Clostridiales;Ruminococcaceae;Oscillibacter	1.770290277	2.054876588	3.828651003	2.181387718	1.756739653	3.101333333
Firmicutes;Clostridia;Clostridiales;Ruminococcaceae;Other	5.903072573	5.492318343	9.064072055	4.99444952	6.064211281	6.305666667
Firmicutes;Clostridia;Clostridiales;Ruminococcaceae;Papillibacter	0.24487963	0.123811806	0.315289534	0.27506803	0.237944564	0.026
Firmicutes;Clostridia;Clostridiales;Ruminococcaceae;Ruminococcus	0.018243182	0.007987858	0.043178592	0.139007594	0.000843775	1.084333333
Firmicutes;Clostridia;Clostridiales;Ruminococcaceae;Sporobacter	-	-	-	-	-	0.0035
Firmicutes;Clostridia;Clostridiales;Ruminococcaceae;Subdoligranulum	0.00841993	-	-	-	0.00168755	4.131
Firmicutes;Clostridia;Clostridiales;Syntrophomonadaceae;Other	-	-	-	-	-	0.000166667
Firmicutes;Clostridia;Clostridiales;Veillonellaceae;Acidaminococcus	-	-	-	-	-	0.046333333
Firmicutes;Clostridia;Clostridiales;Veillonellaceae;Allisonella	-	-	-	-	-	0.020166667
Firmicutes;Clostridia;Clostridiales;Veillonellaceae;Dialister	-	-	-	-	-	6.0445
Firmicutes;Clostridia;Clostridiales;Veillonellaceae;Megamonas	-	-	-	-	-	0.028666667
Firmicutes;Clostridia;Clostridiales;Veillonellaceae;Megasphaera	-	-	0.001004107	-	-	0.411
Firmicutes;Clostridia;Clostridiales;Veillonellaceae;Mitsuokella	-	-	-	-	-	0.263833333
Firmicutes;Clostridia;Clostridiales;Veillonellaceae;Other	-	-	0.005020534	0.000491193	-	0.519166667
Firmicutes;Clostridia;Clostridiales;Veillonellaceae;Phascolarctobacterium	-	-	-	-	-	2.044333333
Firmicutes;Clostridia;Clostridiales;Veillonellaceae;Sporomusa	-	-	-	-	0.000843775	-
Firmicutes;Clostridia;Clostridiales;Veillonellaceae;Succiniclasticum	-	-	0.001004107	-	-	-
Firmicutes;Clostridia;Clostridiales;Veillonellaceae;Veillonella	0.007718269	0.010650478	0.001004107	0.005894315	0.010969076	0.064333333
Firmicutes;Clostridia;Other;Other;Other	0.875672717	0.921931997	0.177726903	0.99712161	0.093659031	1.8725
Firmicutes;Erysipelotrichi;Erysipelotrichales;Erysipelotrichaceae;Allobaculum	-	-	-	-	-	0.000166667
Firmicutes;Erysipelotrichi;Erysipelotrichales;Erysipelotrichaceae;Bulleidia	-	-	-	-	-	0.000166667
Firmicutes;Erysipelotrichi;Erysipelotrichales;Erysipelotrichaceae;Catenibacterium	-	-	-	-	-	0.012833333
Firmicutes;Erysipelotrichi;Erysipelotrichales;Erysipelotrichaceae;Coprobacillus	-	-	-	0.000491193	-	0.005166667
Firmicutes;Erysipelotrichi;Erysipelotrichales;Erysipelotrichaceae;Holdemania	0.002806643	0.005325239	-	0.002455965	-	0.013666667
Firmicutes;Erysipelotrichi;Erysipelotrichales;Erysipelotrichaceae;Other	0.011226573	0.02596054	0.00301232	0.040277819	0.023625701	0.1365
Firmicutes;Erysipelotrichi;Erysipelotrichales;Erysipelotrichaceae;Solobacterium	-	-	-	-	-	0.000333333
Firmicutes;Erysipelotrichi;Erysipelotrichales;Erysipelotrichaceae;Turicibacter	0.018944842	-	-	-	-	0.0095
Firmicutes;Other;Other;Other;Other	0.127702271	0.109167399	0.141579058	0.192547621	0.204193562	0.681666667
Fusobacteria;Fusobacteria;Fusobacteriales;Fusobacteriaceae;Fusobacterium	-	-	-	-	0.000843775	0.001166667
Fusobacteria;Fusobacteria;Fusobacteriales;Fusobacteriaceae;Other	-	-	-	-	-	0.000666667
Fusobacteria;Fusobacteria;Fusobacteriales;Leptotrichiaceae;Leptotrichia	-	-	-	-	-	0.000166667
Lentisphaerae;Lentisphaeria;Victivallales;Victivallaceae;Victivallis	-	-	-	-	-	0.000666667
Other;Other;Other;Other;Other	2.062882844	2.930678398	4.052575032	3.437367992	2.362570139	2.348666667
Planctomycetes;Planctomycetacia;Planctomycetales;Planctomycetaceae;Singulisphaera	-	0.005325239	-	-	-	-
Proteobacteria;Alphaproteobacteria;Caulobacterales;Caulobacteraceae;Caulobacter	-	-	-	-	0.000843775	-
Proteobacteria;Alphaproteobacteria;Other;Other;Other	0.031574737	0.095854301	-	-	-	0.1165
Proteobacteria;Alphaproteobacteria;Rhizobiales;Other;Other	-	0.002662619	-	0.001473579	-	-
Proteobacteria;Alphaproteobacteria;Rhodobacterales;Rhodobacteraceae;Other	-	-	-	0.001964772	-	-
Proteobacteria;Alphaproteobacteria;Rhodobacterales;Rhodobacteraceae;Rhodobaca	-	-	-	0.002455965	-	-
Proteobacteria;Alphaproteobacteria;Rhodobacterales;Rhodobacteraceae;Rhodobacter	-	-	-	0.000491193	-	-
Proteobacteria;Alphaproteobacteria;Rhodospirillales;Other;Other	-	0.00133131	-	-	-	-
Proteobacteria;Betaproteobacteria;Burkholderiales;Alcaligenaceae;Other	0.039293007	0.040604947	-	-	-	0.002
Proteobacteria;Betaproteobacteria;Burkholderiales;Alcaligenaceae;Parasutterella	-	-	0.00301232	0.027997996	0.023625701	0.754333333
Proteobacteria;Betaproteobacteria;Burkholderiales;Alcaligenaceae;Sutterella	-	-	-	-	-	0.191666667
Proteobacteria;Betaproteobacteria;Burkholderiales;Comamonadaceae;Comamonas	-	-	-	-	-	0.000166667
Proteobacteria;Betaproteobacteria;Burkholderiales;Comamonadaceae;Other	-	-	-	0.000982386	-	-
Proteobacteria;Betaproteobacteria;Burkholderiales;Comamonadaceae;Variovorax	-	-	-	0.000491193	-	-
Proteobacteria;Betaproteobacteria;Burkholderiales;Other;Other	-	-	-	-	-	0.059166667
Proteobacteria;Betaproteobacteria;Burkholderiales;Oxalobacteraceae;Other	-	-	-	-	-	0.011166667
Proteobacteria;Betaproteobacteria;Burkholderiales;Oxalobacteraceae;Oxalobacter	-	-	-	-	-	0.002833333
Proteobacteria;Betaproteobacteria;Neisseriales;Neisseriaceae;Microvirgula	-	0.00133131	-	0.000491193	-	-
Proteobacteria;Betaproteobacteria;Other;Other;Other	-	-	-	-	0.000843775	0.004666667
Proteobacteria;Betaproteobacteria;Rhodocyclales;Rhodocyclaceae;Other	-	-	-	0.000982386	-	-
Proteobacteria;Deltaproteobacteria;Desulfovibrionales;Desulfohalobiaceae;Other	-	-	-	-	-	0.000166667
Proteobacteria;Deltaproteobacteria;Desulfovibrionales;Desulfovibrionaceae;Desulfovibrio	-	-	-	-	-	0.109
Proteobacteria;Deltaproteobacteria;Desulfovibrionales;Desulfovibrionaceae;Lawsonia	-	-	-	-	-	0.0035
Proteobacteria;Deltaproteobacteria;Desulfovibrionales;Desulfovibrionaceae;Other	-	-	-	-	-	0.166666667
Proteobacteria;Deltaproteobacteria;Desulfovibrionales;Other;Other	0.176116869	0.184386399	0.413692	0.000491193	0.000843775	0.010333333
Proteobacteria;Deltaproteobacteria;Other;Other;Other	-	0.000665655	0.18174333	0.210230566	-	0.001
Proteobacteria;Epsilonproteobacteria;Campylobacterales;Campylobacteraceae;Arcobacter	-	0.000665655	0.001004107	0.000491193	-	-
Proteobacteria;Epsilonproteobacteria;Campylobacterales;Campylobacteraceae;Campylobacter	-	-	-	-	-	0.001
Proteobacteria;Epsilonproteobacteria;Campylobacterales;Campylobacteraceae;Sulfurospirillum	-	0.000665655	-	-	-	-
Proteobacteria;Epsilonproteobacteria;Campylobacterales;Helicobacteraceae;Sulfurovum	-	-	-	0.000982386	-	-
Proteobacteria;Gammaproteobacteria;Aeromonadales;Aeromonadaceae;Aeromonas	-	-	-	-	-	0.000166667
Proteobacteria;Gammaproteobacteria;Aeromonadales;Aeromonadaceae;Other	-	-	-	-	-	0.000166667
Proteobacteria;Gammaproteobacteria;Aeromonadales;Other;Other	-	-	-	-	-	0.000166667
Proteobacteria;Gammaproteobacteria;Aeromonadales;Succinivibrionaceae;Other	-	-	-	-	-	0.000166667
Proteobacteria;Gammaproteobacteria;Aeromonadales;Succinivibrionaceae;Succinivibrio	-	-	-	-	-	0.408833333
Proteobacteria;Gammaproteobacteria;Enterobacteriales;Enterobacteriaceae;Buttiauxella	-	-	-	-	-	0.000166667
Proteobacteria;Gammaproteobacteria;Enterobacteriales;Enterobacteriaceae;Citrobacter	-	-	-	0.005894315	0.00843775	0.000166667
Proteobacteria;Gammaproteobacteria;Enterobacteriales;Enterobacteriaceae;Escherichia/Shigella	0.007718269	-	-	0.005894315	0.007593975	0.173833333
Proteobacteria;Gammaproteobacteria;Enterobacteriales;Enterobacteriaceae;Morganella	0.000701661	-	-	0.000491193	-	-
Proteobacteria;Gammaproteobacteria;Enterobacteriales;Enterobacteriaceae;Other	0.004209965	0.009319168	0.002008214	0.010806244	0.00843775	0.023833333
Proteobacteria;Gammaproteobacteria;Enterobacteriales;Enterobacteriaceae;Proteus	-	-	-	-	0.010969076	-
Proteobacteria;Gammaproteobacteria;Legionellales;Legionellaceae;Tatlockia	-	-	-	-	-	0.000166667
Proteobacteria;Gammaproteobacteria;Other;Other;Other	-	-	-	0.002455965	0.004218875	0.008666667
Proteobacteria;Gammaproteobacteria;Pasteurellales;Pasteurellaceae;Aggregatibacter	-	-	-	-	-	0.000166667
Proteobacteria;Gammaproteobacteria;Pasteurellales;Pasteurellaceae;Haemophilus	-	-	-	-	-	0.0435
Proteobacteria;Gammaproteobacteria;Pasteurellales;Pasteurellaceae;Other	-	-	-	-	-	0.012
Proteobacteria;Gammaproteobacteria;Pseudomonadales;Moraxellaceae;Acinetobacter	0.002104982	0.005325239	-	0.004420736	0.004218875	0.000333333
Proteobacteria;Gammaproteobacteria;Pseudomonadales;Moraxellaceae;Enhydrobacter	0.000701661	-	-	0.000982386	-	-
Proteobacteria;Gammaproteobacteria;Pseudomonadales;Moraxellaceae;Other	-	-	-	-	-	0.000166667
Proteobacteria;Gammaproteobacteria;Xanthomonadales;Xanthomonadaceae;Other	-	-	-	0.000491193	-	-
Proteobacteria;Gammaproteobacteria;Xanthomonadales;Xanthomonadaceae;Stenotrophomonas	-	-	-	0.000491193	-	-
Proteobacteria;Other;Other;Other;Other	-	0.00133131	1.402737195	0.62430619	-	0.016666667
Spirochaetes;Spirochaetes;Spirochaetales;Brachyspiraceae;Brachyspira	-	-	-	-	-	0.001666667
Synergistetes;Synergistia;Synergistales;Synergistaceae;Cloacibacillus	-	-	-	-	-	0.009
Synergistetes;Synergistia;Synergistales;Synergistaceae;Jonquetella	-	-	-	-	-	0.000833333
Synergistetes;Synergistia;Synergistales;Synergistaceae;Other	-	-	-	-	-	0.000166667
Tenericutes;Mollicutes;Anaeroplasmatales;Anaeroplasmataceae;Anaeroplasma	0.015436538	0.089197753	0.016065709	0.005403122	0.264945366	-
Tenericutes;Mollicutes;Anaeroplasmatales;Anaeroplasmataceae;Asteroleplasma	-	-	-	-	-	0.2855
TM7;TM7_genera_incertae_sedis;Other;Other;Other	0.097530856	0.118486567	0.700866544	0.223001582	0.046407628	0.000666667
Unclassified;Other;Other;Other;Other	-	-	-	-	-	0.0105
Verrucomicrobia;Opitutae;Other;Other;Other	-	-	-	-	-	0.000166667
Verrucomicrobia;Opitutae;Puniceicoccales;Puniceicoccaceae;Other	-	-	-	-	-	0.016666667
Verrucomicrobia;Other;Other;Other;Other	-	-	-	-	-	0.000166667
Verrucomicrobia;Spartobacteria;Spartobacteria_genera_incertae_sedis;Other;Other	-	0.00133131	-	-	-	-
Verrucomicrobia;Verrucomicrobiae;Verrucomicrobiales;Verrucomicrobiaceae;Akkermansia	-	1.843863993	-	-	6.415221702	4.450666667

The relative distribution of the gut microbial genera among single human (16 individuals) and five mouse groups (15, 14, 19, 19, 21 mice respectively).
The abundance threshold within at least one of the category was set to 0.19%.

legend
0-0.19%
>0.19-2%
>2-4%
>4-8%
>8-16%
>16%
